# The Novel Imiqualine EAPB02303 Is a Potent Drug for Treating Acute Myeloid Leukemia

**DOI:** 10.3390/biom15050741

**Published:** 2025-05-20

**Authors:** Perla Makhoul, Rita Hleihel, Shaymaa Itani, Maguy Hamie, Stephanie Pagniagua-Gayraud, Cindy Patinote, Myriam Richaud, Raghida Abou Merhi, Marwan El-Sabban, Simon Galas, Carine Deleuze-Masquefa, Pierre-Antoine Bonnet, Hiba El Hajj

**Affiliations:** 1 Institut des Biomolécules Max Mousseron (IBMM), UMR 5247, CNRS, ENSCM, Université de Montpellier, 34090 Montpellier, France or pm31@aub.edu.lb (P.M.); stephanie.paniagua-gayraud@umontpellier.fr (S.P.-G.); cindy.patinote@umontpellier.fr (C.P.); myriam.richaud@umontpellier.fr (M.R.); simon.galas@umontpellier.fr (S.G.); carine.masquefa@umontpellier.fr (C.D.-M.); pierre-antoine.bonnet1@umontpellier.fr (P.-A.B.); 2 Department of Biology, Faculty of Sciences, GSBT Laboratory, Lebanese University, R. Hariri Campus, Hadath 1533, Lebanon; raboumerhi@ul.edu.lb; 3 Department of Experimental Pathology, Immunology and Microbiology, Faculty of Medicine, American University of Beirut, Beirut P.O. Box 113-6044, Lebanon; ski02@mail.aub.edu (S.I.); mh242@aub.edu.lb (M.H.); 4 Department of Internal Medicine, Faculty of Medicine, American University of Beirut, Beirut P.O. Box 113-6044, Lebanon; rh150@aub.edu.lb; 5 Department of Anatomy, Cell Biology and Physiological Sciences, Faculty of Medicine, American University of Beirut, Beirut P.O. Box 113-6044, Lebanon; me00@aub.edu.lb

**Keywords:** acute myeloid leukemia, imiqualines, PI3K/AKT/mTOR, NPM1c

## Abstract

Although 60% of AML patients respond well to standard chemotherapy, most patients eventually relapse, develop chemoresistance, and do not survive more than five years. Targeted therapies, including analogs of imiquimod belonging to the family of imiqualines, emerged as promising agents against AML. Notably, the first-generation imiqualine EAPB0503 proved selective potency against nucleophosmin-1-mutant (NPM1c) AML. Recently, chemical modifications of EAPB0503 led to the development of the lead compound from the second generation, EAPB02303. Here, we demonstrate that EAPB02303 displays 200-fold greater potency, broader activity across AML subtypes, and, importantly, a distinct mechanistic profile when compared to EAPB0503. Unlike EAPB0503, which primarily targeted *NPM1c* AML cells, EAPB02303 exhibits broad-spectrum activity across various AML subtypes. Remarkably, EAPB02303 anti-leukemic activity was attributed to the inhibition of PI3K/AKT/mTOR signaling activity. Nevertheless, NPM1c AML cells were more sensitive to EAPB02303, likely due to its ability to promote NPM1c protein degradation. In vivo, EAPB02303 potently reduced the leukemic burden and improved organ tumor infiltration in both wt-*NPM1* and *NPM1c* AML xenograft mice. Yet, the significant prolonged survival was exclusive to *NPM1c* AML xenografts, likely due to superior response conferred by NPM1c degradation. Overall, these findings highlight the potential of EAPB02303 as a powerful therapeutic agent for a range of AML subtypes, supporting its further development for broader clinical use.

## 1. Introduction

Acute myeloid leukemia (AML) is a heterogeneous and highly complex hematological malignancy, characterized by an abnormal proliferation and differentiation of hematopoietic stem cells and myeloid progenitor cells [1], leading to BM failure. AML is classified as the most common leukemia among adults, accounting for 80% of all cases [2,3], with an estimated 5-year relative survival of 30% [4].

The combination of cytarabine (AraC) with an anthracycline known as the “7 + 3” regimen remained the standard of care for AML patients fit for chemotherapy for decades [5]. In total, 60% of AML patients respond well to this combination [6], yet most patients eventually relapse, develop chemoresistance, and do not survive for more than 5 years [7]. In newly diagnosed patients ineligible for intensive chemotherapy, the BCL2 inhibitor venetoclax combined with hypomethylating agents demonstrated favorable clinical responses [8,9]. Yet, this combination is associated with high risks of infectious complications, toxicity, and variable efficacy across different AML subtypes [8,10,11].

It is now well-established that recurrent mutations determine both the prognosis and the therapeutic management of AML patients [6,12]. Mutations resulting in constitutive activation of signaling pathways such as the PI3K/AKT/mTOR pathway are essential for the survival of AML blasts [13]. Indeed, PI3K/AKT/mTOR hyperactivation is reported in 60% of AML cases, associating with poor response and decreased overall survival [14,15,16,17,18]. Moreover, targeted therapies directed against the PI3K/AKT/mTOR pathway, either alone or in combination with chemotherapeutic agents, have significantly contributed to overcoming treatment resistance in AML [19,20,21,22,23].

*NPM1* mutations account for 30% of AML cases [1,24]. *NPM1* is a multifunctional phosphoprotein that continuously shuttles between the cytoplasm, nucleus, and nucleolus [1,24]. Two main proteins, a SUMO-specific peptidase 3 (SENP3) and the ADP-ribosylation factor (ARF), are involved in the posttranslational modifications of *NPM1* [25]. SENP3 activates ribosomal biogenesis through *NPM1* de-SUMOylation, while ARF prohibits it by SUMOylating NPM1 [25]. Mutations in *NPM1* result in the cytoplasmic translocation of the mutant protein (NPM1c), contributing to AML leukemogenesis [26]. We and others demonstrated the efficacy of targeting NPM1c for the treatment of AML [27,28,29].

Imiqualines are analogs of imiquimod [30] with proven potency against hematopoietic malignancies [29,31,32,33]. EAPB0503, belonging to the first-generation imiqualines, is selectively active against *NPM1c* AML in vitro and in vivo [29,32]. Indeed, EAPB0503 conferred a significant prolonged survival and lessened leukemia burden exclusively in *NPM1c* AML xenograft mice [29,32]. Chemical modifications of the first-generation imiqualines led to the synthesis of the lead compound of the second-generation derivative EAPB02303 [34]. EAPB02303 surpassed EAPB0503 potency against melanoma [35].

Here, we showed that EAPB02303 induces cell growth arrest and apoptosis at concentrations 200-fold lower than EAPB0503 in both *NPM1c* and wt-*NPM1* AML. At the molecular level, EAPB02303 potently inhibits the PI3K/AKT/mTOR pathway, underlying its robust anti-leukemic activity. Nevertheless, *NPM1c* AML cells were more sensitive to EAPB02303 than the other tested cell lines, with a notable degradation of NPM1c, concomitant with downregulation of SENP3 and upregulation of ARF. Finally, EAPB02303 drastically lowered the leukemic burden in the BM of both *NPM1c* and wt-*NPM1* xenograft mice and significantly reduced AML-associated hepatosplenomegaly in all treated animals. Yet, the conferred advantage of survival was only obtained in *NPM1c* xenograft animals.

## 2. Materials and Methods

### 2.1. Cell Lines and Primary Cells

OCI-AML2 and OCI-AML3 cells were grown in MEM-α supplemented with 20% fetal bovine serum (FBS) and 1% penicillin-streptomycin. MOLM-13, THP-1, and KG-1α cells were grown in RPMI-1640 supplemented with 10% FBS and 1% penicillin-streptomycin. Primary blasts from AML patients were collected after approval by the Institutional Review Board at the American University of Beirut (AUB) and after patients provided informed consent in accordance with the Declaration of Helsinki (IRB ID #IM.AB.29). Patients’ characteristics are summarized in Table 1. Healthy human PBMCs were isolated following the Ficoll separation (17144002 Ficoll-Paque PLUS Cytiva) from blood samples provided by the AUB Medical Center. Cells were seeded at a density of 2 × 10^5^/mL and treated with increasing concentrations of EAPB02303. Cell viability was assessed at 24 h, 48 h, and 72 h using the trypan blue exclusion dye.

### 2.2. Drugs

EAPB02303 synthesis and purity for biological studies were performed as previously described [35]. EAPB02303 was dissolved in dimethylsulfoxide (DMSO) at a concentration of 10^−2^ M, aliquoted, and stored at −20 °C. EAPB02303 was used at a final concentration of 5 nM (OCI-AML2, OCI-AML3, MOLM-13, and primary blasts from AML patients), 10 nM (KG-1α), or 100 nM (THP-1).

### 2.3. Cell Cycle Analysis

Cells treated with EAPB02303 for 24 h and 48 h were harvested, washed with PBS, fixed with 100% cold ethanol, and stored at −20 °C. At least 24 h later, cells were rewashed with PBS and then treated for 45 min with 100 μL of 200 μg/mL DNase-free RNase A (EN0531, Thermo Fischer Scientific, Waltham, MA, USA). Cell pellets were then resuspended in 500 μL PBS and were stained with 30 μL of 1 mg/mL propidium iodide (CAS25535-16-4, Sigma-Aldrich, Darmstadt, Germany) and incubated for 10 min at room temperature in the dark. Data were analyzed on the Guava Easycyte 8 flow cytometer.

### 2.4. Annexin V/PI Assay

Cells were treated with EAPB02303 as previously described before Annexin V labeling. An annexin V-FLUOS staining kit (11988549001, Roche, Basel Switzerland) was used to assess phosphatidylserine exposure and cell viability, according to the manufacturer’s instructions. Briefly, cells were collected at 24 h and 48 h and washed with PBS. Each sample was resuspended in 100 μL of Annexin-V-FLUOS incubation buffer and then labeled with 2 μL of Annexin-V-FLUOS labeling reagent. Samples were incubated at room temperature in the dark for 15 min and then analyzed on a Guava Easycyte 8 flow cytometer.

### 2.5. Immunoblotting

AML cells treated with EAPB02303 as previously described were collected at 6 h, 24 h, and 48 h post-treatment. Proteins were extracted using Laemmli lysis buffer and denatured at 95 °C for 10 min, separated by SDS-PAGE (1 h, 100 V), and then transferred onto nitrocellulose membranes (overnight transfer, 30 V). The membranes were blocked (5% skimmed milk in PBS) for 1 h under agitation and then probed overnight at 4 °C with the primary antibodies summarized in Table 2. The following day, blots were washed and then incubated with the corresponding horseradish peroxidase (HRP)-conjugated secondary antibodies for 1 h at room temperature. Proteins were detected using the luminol detection kit (Clarity Western ECL substrate Bio-Rad, Hercules, CA, USA), and images were captured using the Bio-Rad Chemidoc MP system (Image Lab Software 5.0).

### 2.6. Xenograft Animal Studies

This animal study was reviewed and approved by the Institutional Animal Care and Utilization Committee of the AUB (IACUC approval #24-09-632). NOD/Shi-*scid IL2r-gamma*^−/−^ (NSG) male and female mice were obtained from Jackson Laboratories (Bar Harbor, ME, USA). Moreover, 2 × 10^6^ OCI-AML2 or OCI-AML3 cells were injected into the tail vein of 6–8-week-old mice. One week later, mice were treated intraperitoneally with EAPB02303 every other day over 3 weeks at a dose of 2.5 mg/kg/day. EAPB02303 was dissolved in DMSO and diluted in lipofundin before administration. A group of mice per condition was monitored for survival. Another group was used to assess the efficacy of the drug on tumor burden, spleen weight, and gross and histological features of the livers and the spleen. To assess leukemic burden in mice, human CD45 staining was performed. Bone marrow cells were flushed from the femurs and tibias of euthanized animals. Cell surface staining was performed on 100 µL of the sample using 10 µL of anti-human CD45 PE antibody (#555483, BD Pharmingen, Franklin Lakes, NJ, USA). After incubation for 15 min in the dark, samples were analyzed on the BD FACS Aria cell sorter. Livers and spleens from untreated controls or EAPB02303-treated mice were fixed in neutral buffer formalin, embedded in paraffin, sectioned, and stained with hematoxylin and eosin (H&E). Histopathology was examined using the Olympus CX41 microscope (Tokyo, Japan).

### 2.7. Statistical Analysis

Data were run in at least three independent experiments and reported as the average ± standard deviations (SDs). Statistical analysis was performed using two-way ANOVA or Student’s *t*-test. A *p*-value of equal to or less than 0.05 was considered statistically significant.

## 3. Results

### 3.1. EAPB02303 Abrogates Cell Proliferation of AML Cells with No Cytotoxicity on Human Peripheral Blood Mononuclear Cells

**Figure 1:**
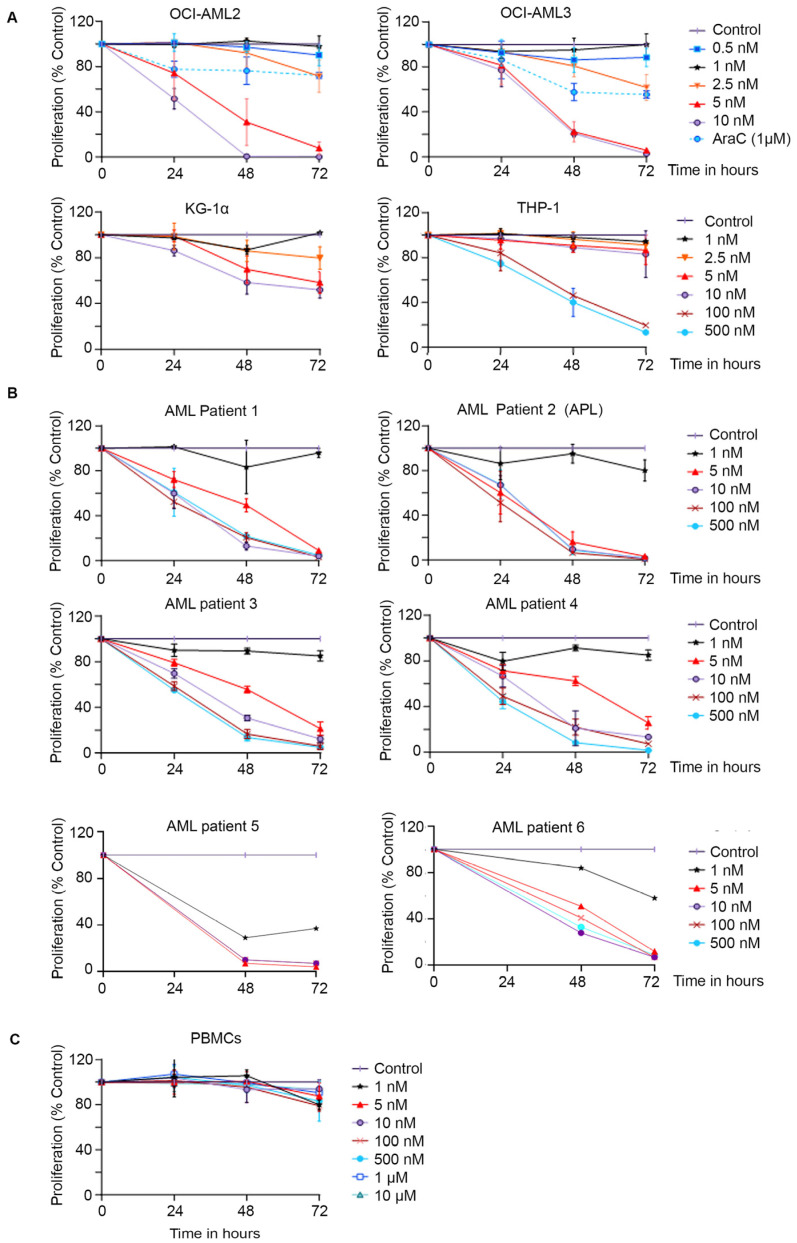
EAPB02303 induces growth inhibition in acute myeloid leukemia (AML) cell lines and primary blasts derived from AML patients. (**A**) AML cell lines (OCI-AML2, OCI-AML3, KG-1α, and THP-1) were treated with increasing concentrations of EAPB02303 for 24, 48, and 72 h. Cytarabine (AraC) was used as a positive control for OCI-AML2 and OCI-AML3. (**B**) Primary blasts derived from AML patients (Patients 1 and 6: *NPM1c*, patient 2: acute promyelocytic leukemia (APL) with chromosomal translocation t(15; 17), patient 3: inversion INV(16), patient 4: *DNMT3A/IDH2/TET2* mutations, and P5 expressed wt-NPM1/IKZF1/PTPN1/RUNX1) were treated with increasing concentrations of EAPB02303, as previously described. (**C**) Human peripheral blood mononuclear cells (PBMCs) from healthy patients were treated with EAPB02303, as described previously. AML cellular proliferation was assessed using the trypan blue exclusion assay (percentage of the untreated control). The results shown represent the average of at least 3 independent experiments ± SD. Two-way ANOVA was performed to validate significance as compared to the untreated control and is detailed in Supplementary Table S2.

We previously demonstrated the selective activity of EAPB0503 against *NPM1c* AML cell lines and primary blasts from *NPM1c* AML patients [29,32]. Here, we evaluated the anticancer effect of EAPB02303 on AML cell viability. AraC was used as a positive control at a final concentration of 1 µM. EAPB02303 inhibited cell growth in a concentration- and time-dependent manner, and in all tested AML cells (Figure 1). In both wt-*NPM1* OCI-AML2 and *NPM1c* OCI-AML3, a concentration as low as 5 nM of EAPB02303 resulted in complete growth inhibition 72 h post-treatment (*p* < 0.001) (Figure 1A). This concentration was prominently better than the positive control AraC, which only resulted in growth inhibition by 20% and 50% in OCI-AML2 and OCI-AML3 at 72 h (*p* < 0.01 and *p* < 0.001, respectively) (Figure 1A). EAPB02303 at a higher dose of 10 nM displayed a comparable time-dependent decrease in cell proliferation of OCI-AML3, reaching 80% inhibition at 48 h (*p* < 0.001) and complete cell growth abrogation at 72 h (*p* < 0.001). The same dose of 10 nM potently induced a 50% reduction in OCI-AML2 cell viability starting at 24 h (*p* < 0.001) and entirely suppressed cell growth starting at 48 h (*p* < 0.001) (Figure 1A). In MOLM-13 cells, EAPB02303 significantly reduced cell viability by more than 60% at 24 h (*p* < 0.01) to completely abolish it at 48 h (*p* < 0.001) at the low dose of 5 nM (Supplementary Figure S1). Strikingly, in AML cells where P53 is affected (KG-1α and THP-1) and known to be resistant to drugs [36,37], EAPB02303 proved efficient and resulted in partial or complete cell growth inhibition. Indeed, KG-1α exhibited a median decrease in cell proliferation at 72 h following treatment with 10 nM EAPB02303 (*p* < 0.001) (Figure 1A). Lastly, in *TP53*-null THP-1, while concentrations of EAPB02303 ranging from 1 nM to 10 nM displayed no anti-growth effect, 100 nM and up to 500 nM of the molecule potently reduced cell viability to 50% 48 h post-treatment (*p* < 0.001), with near complete inhibition of proliferation at 72 h (*p* < 0.001) (Figure 1A).

We then assessed the activity of EAPB02303 on primary leukemic blasts derived from AML patients (patient 1 and 6 harbored *NPM1* mutation, patient 2 had acute promyelocytic leukemia (APL), patient 3 harbored inversion INV(16), patient 4 expressed DNMT3A/IDH2/TET2/EZH2 mutations, and P5 expressed wt-NPM1/IKZF1/PTPN1/RUNX1) (AML patients’ information is detailed in Table 1). All tested primary blasts from AML patients were sensitive to treatment with 5 nM EAPB02303 starting 24 h (*p* < 0.001) (Figure 1B). After 48 h, a sharp decrease in proliferation was observed in blasts from patients 1, 3, and 4 (~50% growth inhibition, *p* < 0.001), while a complete suppression of cellular growth of APL cells from patients 2 and 5 was achieved (*p* < 0.001) (Figure 1B). Lastly, EAPB02303 completely abolished cell viability in all tested primary blasts at 72 h (*p* < 0.001) (Figure 1B). Importantly, EAPB02303 displayed no growth-inhibitory effect on healthy human PBMCs up to 72 h of treatment, even at the highest concentration of 10 µM (Figure 1C).

Overall, our findings demonstrate that EAPB02303 strongly inhibits the proliferation of an array of AML cells at nanomolar doses while showing no toxic effects on normal human PBMCs.

### 3.2. EAPB02303 Induces Cell Cycle Arrest and Cell Death in AML Cells

**Figure 2:**
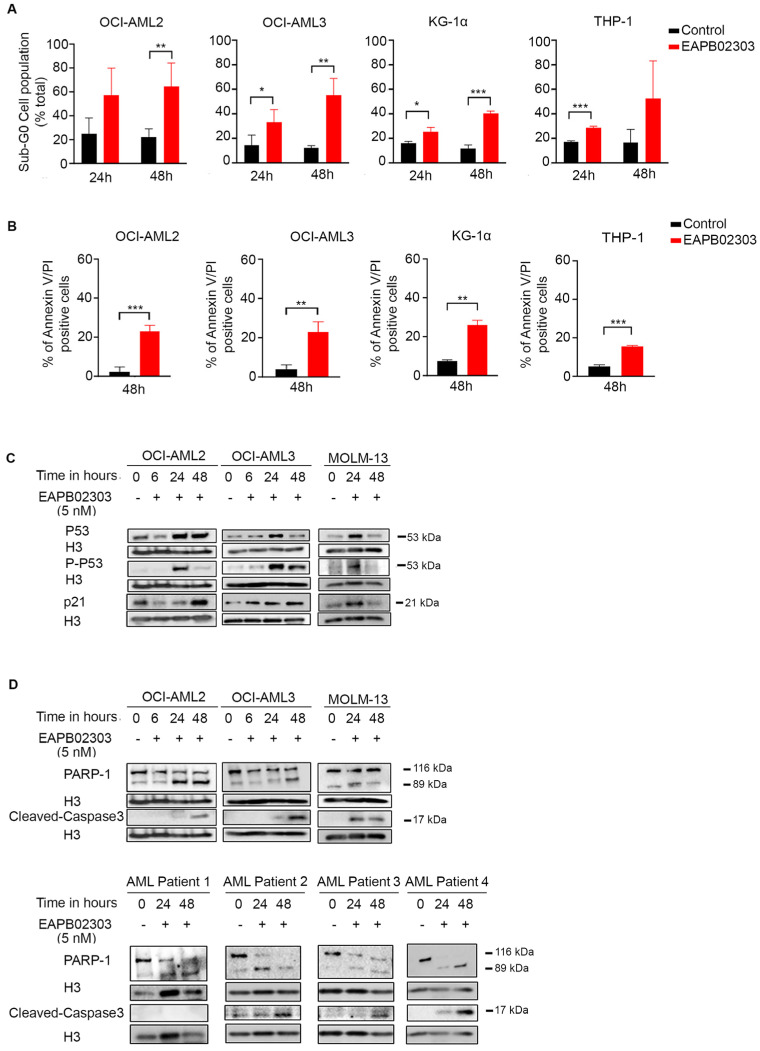
EAPB0203 induces P53-mediated apoptosis in AML cells. (**A**) Cell cycle analysis after PI staining of OCI-AML2 and OCI-AML3, following treatment with 5 nM EAPB02303, and KG-1α and THP-1 treated with 10 nM or 100 nM, respectively, at 24 and 48 h. Histograms represent the percentage of the sub-G0 cell population. (**B**) Annexin V/PI staining of OCI-AML2, OCI-AML3, KG-1α, and THP-1 cells upon treatment with EAPB02303 as previously described. Histograms represent the percentage of the AnV/PI-positive cell population. (**C**) Western blot analysis of pro-apoptotic proteins P53, P-P53, P21, and H3 in OCI-AML2, OCI-AML3, and MOLM-13 cells treated with 5 nM EAPB02303. (**D**) Western blot analysis of PARP-1 and cleaved caspase 3 in OCI-AML2, OCI-AML3, and MOLM-13 cell lines and AML patient 1 (NPM1c), AML patient 2 (APL), AML patient 3 (INV16), and AML patient 4 (*DNMT3A/IDH2/TET2/EZH2* mutations) cells treated with 5 nM EAPB02303. * *p* < 0.05; ** *p* < 0.01; *** *p* < 0.001. All original western blots can be found at Supplementary Materials.

To explore the molecular mechanism of EAPB02303, we adopted the dose of 5 nM on OCI-AML2, OCI-AML3, or MOLM-13 cell lines. In the more resistant cells, KG-1α and THP-1, 10 nM and 100 nM, were selected, respectively. EAPB02303 significantly increased the sub-G0 cell population in all treated AML cells after 24 h treatment (OCI-AML3 and KG-1α *p* < 0.05, THP-1 *p* < 0.001) (Figure 2A). A sharper and more significant increase in sub-G0 population was observed 48 h post-treatment with EAPB02303, reaching 60% in both OCI-AML2 and OCI-AML3 cells (*p* < 0.01) and around 40% in both KG-1α (*p* < 0.001) and THP-1 (Figure 2A), without any noticed variation in the cell cycle distribution (Supplementary Figure S2A,B), demonstrating a main effect of EAPB02303 on the sub-G0 accumulation.

We then assessed apoptosis using Annexin V/PI and demonstrated a significant increase in Annexin V/PI positivity in OCI-AML2 (*p* < 0.001), OCI-AML3 (*p* < 0.01), KG-1α (*p* < 0.01), and THP-1 (*p* < 0.001), 48 h post-treatment (Figure 2B and Supplementary Figure S2C). These results confirm the induction of late apoptosis in all tested AML cells.

Consistent with the Annexin V/PI data, EAPB02303 induces an upregulation of P53 and its active phosphorylated form P-P53 in the *NPM1c*-expressing OCI-AML3 in a time-dependent manner and starting 6 h post-treatment (Figure 2C and Supplementary Figure S3A). Interestingly, a significant increase in P53 and P-P53 levels was also obtained, only at 24 h, in the wt-*NPM1*-expressing OCI-AML2 (*p* < 0.05) and MOLM-13 (Figure 2C). A time-dependent upregulation of the downstream effector P21 was likewise denoted, starting 6 h following treatment in OCI-AML3, to reach its peak at 48 h (*p* < 0.01) (Figure 2C and Supplementary Figure S3B). In MOLM-13, the significant increase in P21 levels was observed at 24 h post-treatment (*p* < 0.05), while in OCI-AML2, it was only achieved at 48 h (*p* < 0.05) (Figure 2C and Supplementary Figure S3B).

Moreover, EAPB02303 induced a caspase-dependent apoptosis, as demonstrated by the cleavage of procaspase 3 into its active form starting 24 h in MOLM-13, KG-1α, and THP-1 and at 48 h in OCI-AML2 and OCI-AML3 (*p* < 0.01 and *p* < 0.05, respectively) (Figure 2D and Supplementary Figure S3C,D). PARP-1 was also cleaved into its active form starting 24 h in OCI-AML2 and MOLM-13 (*p* < 0.01 and *p* < 0.05, respectively) and at 48 h following treatment with EAPB02303 in OCI-AML3 (Figure 2D and Supplementary Figure S3E). Similarly, treatment of primary blasts derived from AML patients with EAPB02303 revealed PARP-1 and caspase 3 degradation, highlighting the significant EAPB02303-orchestrated apoptotic cell death of AML ex vivo (Figure 2D).

Altogether, these results demonstrate that EAPB02303 induces apoptosis in all tested AML cells.

### 3.3. The Mechanism of Action of EAPB02303 Involves the PI3K/AKT/mTOR Signaling

**Figure 3:**
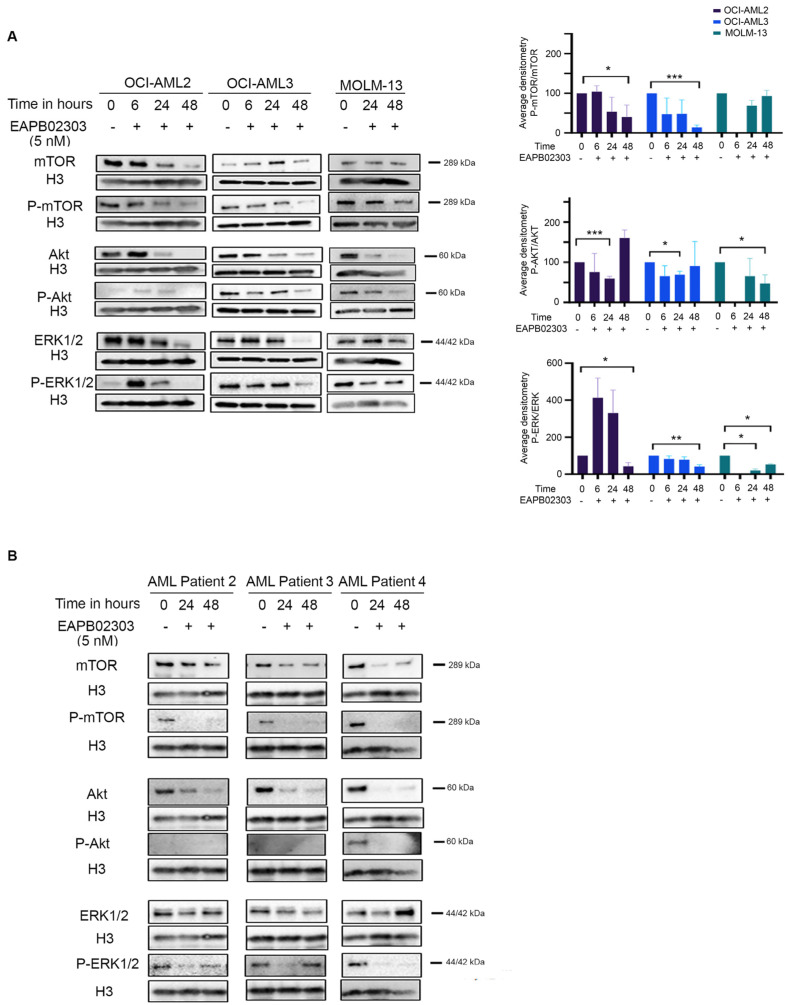
EAPB02303 signals through inhibition of the PI3K/AKT/mTOR pathway in AML. (**A**) Western blot analysis of mTOR, P-mTOR, AKT, P-AKT, ERK, P-ERK, and H3 in OCI-AML2, OCI-AML3, and MOLM-13 cells treated with 5 nM EAPB02303. (**B**) Western blot analysis of mTOR, P-mTOR, AKT, P-AKT, ERK, P-ERK, and H3 in AML patient 2 (APL), AML patient 3 (INV16), and AML patient 4 (*DNMT3A/IDH2/TET2* mutations) cells treated with 5 nM EAPB02303. * *p* < 0.05; ** *p* < 0.01; *** *p* < 0.001.

For most AML patients, continuous activation of the PI3K/AKT/mTOR signaling pathway results in a constitutive phosphorylation and activation of AKT at Ser473 by mTORC2 [38,39]. This dysregulation is indispensable for AML cell survival [13,17] and is seen in 60% of AML cases [17,40]. We recently demonstrated that EAPB02303 downregulates the PI3K/AKT signaling activity in the nematode model *Caenorhabditis elegans* [41]. Consistently, EAPB02303 decreased the protein expression levels of AKT and the phosphorylated AKT at Ser473 at 24 h in OCI-AML2 and OCI-AML3 (*p* < 0.001 and *p* < 0.05, respectively). This was paralleled with a significant decrease in P-AKT levels in MOLM-13 and KG-1α (*p* < 0.05 and *p* < 0.001, respectively) and AKT in THP-1 starting at 24 h (Figure 3A and Supplementary Figure S4A,B), while mTOR levels remained unchanged in MOLM-13, KG-1α, and THP-1 (Supplementary Figure S4A,C). Nevertheless, EAPB02303 treatment resulted in a downregulation of mTOR and its phosphorylated form P-mTOR in both OCI-AML2 and OCI-AML3 cells starting at 24 h. Importantly, EAPB02303 significantly reduced the ratio P-mTOR/mTOR to 40% (*p* < 0.05) and 14% (*p* < 0.001) at 48 h, respectively (Figure 3A and Supplementary Figure S4C). These observations support that EAPB02303’s mechanism of action implicates the PI3K/AKT/mTOR molecular pathway in AML cell lines.

Crosstalk between PI3K/AKT/mTOR and the mitogen-activated protein kinase (MAPK) pathway was previously reported [42]. Our recent findings suggest that EAPB02303 simultaneously reduces their signaling activity [41]. EAPB02303 treatment attenuates the expression levels of ERK and its phosphorylated form in both OCI-AML2 and OCI-AML3 at 48 h and reduces the ratio of P-ERK/ERK to reach 42% in OCI-AML2 and 33% in OCI-AML3 (*p* < 0.05 and *p* < 0.01, respectively) (Figure 3A and Supplementary Figure S4D). Interestingly, in MOLM-13, KG-1α, and THP-1 cell lines, EAPB02303 significantly decreased ERK phosphorylation as early as 24 h, to reach 19%, 39%, and 42%, respectively (*p* < 0.05). This decrease was also sustained up to 48 h (*p* < 0.01) (Figure 3A and Supplementary Figure S4A,D).

Finally, AML patients exhibited a similar protein expression profile, with a decrease in AKT, mTOR, and ERK proteins and their phosphorylated forms starting 24 h and up to 48 h post-treatment with EAPB02303 (Figure 3B).

Our results support a novel molecular mechanism of EAPB02303 in AML through the dual inhibition of PI3K/AKT/mTOR and MAPK pathways.

### 3.4. EAPB02303 Induces NPM1c Degradation, Attenuates SENP3 Expression, and Enhances ARF Levels in NPM1c OCI-AML3

**Figure 4:**
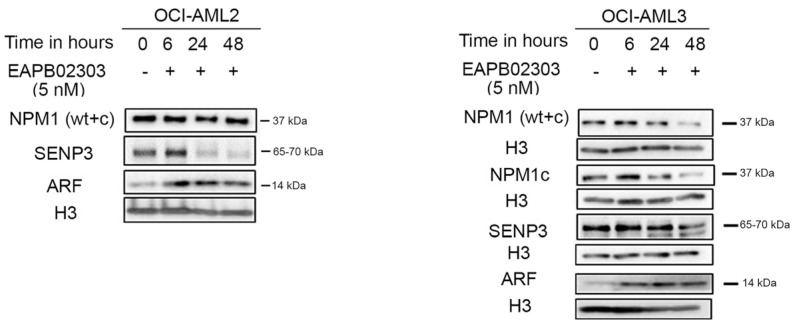
EAPB02303 degrades the mutant protein NPM1c in the OCI-AML3 cell line . Western blot analysis of NPM1 (wt + c), SENP3, ARF, and H3 in OCI-AML2 and OCI-AML3 cells and NPM1c in OCI-AML3, treated with EAPB02303, as described previously.

Despite the unexclusive potency of EAPB02303 against all tested AML profiles, we noticed a higher potency of this drug in cells expressing NPM1c. We demonstrated that EAPB02303 remarkably decreased NPM1c protein levels in OCI-AML3 cells, reaching less than 12% at 48 h post-treatment (*p* < 0.001) (Figure 4, Supplementary Figure S5A,B). Importantly, this was concomitant with a gradual attenuation of SENP3 levels (*p* < 0.01 48 h post-treatment) and increased ARF levels (Figure 4, Supplementary Figure S5C,D). This result indicates that EAPB02303 exhibits similar molecular activity on the SENP3/NPM1/ARF pathway as its parental compound, EAPB0503, in *NPM1c* AML cells, but at 200-fold lower concentrations [29].

### 3.5. EAPB02303 Exhibits Potent In Vivo Activity Against AML

**Figure 5:**
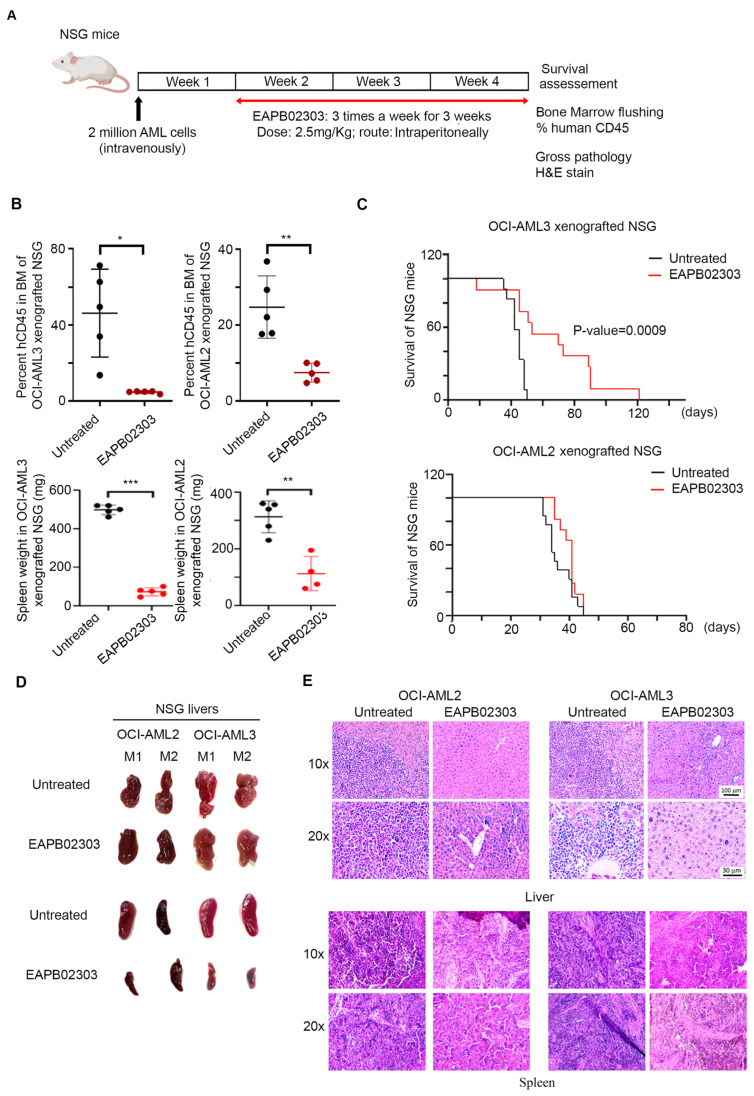
EAPB02303 exhibits in vivo potency on AML xenograft mice. (**A**) Six-to-eight-week-old NSG mice were injected with 2 million OCI-AML2 or OCI-AML3 cells intravenously. One week post-injection, EAPB02303 (2.5 mg/kg) was intraperitoneally administered every other day for 3 weeks. At the end of week 3, mice were sacrificed for hCD45 staining on the flushed bone marrow cells. (**B**) Graph showing the percentage of hCD45 in the BM and spleen weight of OCI-AML2 and OCI-AML3 xenograft mice treated or not with EAPB02303 (n = 5 mice per condition, per cell line). (**C**) Kaplan–Meier overall survival of untreated NSG mice injected with OCI-AML2 (n = 13) or OCI-AML3 (n = 12) (black line) or treated with EAPB02303 (n = 11 per cell line, red line). *p*-values equal to or less than 0.05 were considered significant. (**D**) Gross pathology of livers and spleens from two representative untreated or EAPB02303-treated OCI-AML2 and OCI-AML3 xenografts. (**E**) Histological analysis (H&E stain) of the liver of one representative untreated or EAPB02303-treated OCI-AML2 and OCI-AML3 xenograft mouse. Images were taken at 10× and 20× magnification. * *p* < 0.05; ** *p* < 0.01; *** *p* < 0.001.

We then explored the in vivo efficacy of EAPB02303 (timeline described in Figure 5A). We closely monitored treated animals for signs of distress, weight loss, or behavioral changes and observed no overt toxicity at the tested dose and regimen. Leukemic burden in the BM significantly decreased from 46% to only 5% (*p* < 0.05), and spleen weight drastically decreased from 498 mg to 90 mg (*p* value < 0.0001) upon treatment of OCI-AML3 xenograft mice with EAPB02303 (Figure 5B). Interestingly, EAPB02303 remarkably reduced OCI-AML2 leukemic burden in the BM of xenograft mice from 25% to 8% (*p* < 0.01) and significantly reduced spleen weight from 313 mg to 112 mg (*p* value = 0.0014) (Figure 5B). We then interrogated the impact of EAPB02303 on the potency of EAPB02303 on the survival of AML xenografts. While untreated OCI-AML3 control mice succumbed at day 50 (n = 12 mice), EAPB02303 significantly prolonged the survival of treated xenografts for up to 120 days (n = 11 mice, *p* < 0.001) (Figure 5C). Surprisingly, and despite the potent activity of EAPB02303 on OCI-AML2 in vitro and on leukemic burden in vivo, both OCI-AML2 xenografted mice, whether untreated (n = 13 mice) or treated with EAPB02303 (n = 11 mice), succumbed at day 45 (Figure 5C).

We then examined the liver and spleen gross pathology in OCI-AML2 and OCI-AML3 xenograft mice following treatment with EAPB02303. While untreated control mice displayed white nodules in the liver and marked spleen enlargement, EAPB02303 showed a normal gross macroscopy in the livers and the spleens of OCI-AML2 and OCI-AML3 xenografts (Figure 5D). Consistent with these observations, H&E stain showed a clear infiltration of the liver and the spleen, masking its normal white and red pulp in untreated xenograft mice injected with OCI-AML2 or OCI-AML3, while treatment with EAPB02303 preserved the normal architecture of this organ (Figure 5E).

Taken together, our data highlight the strong in vivo anti-leukemic efficacy of EAPB02303 across both tested AML subtypes while revealing a distinct therapeutic survival advantage in NPM1c-driven AML.

## 4. Discussion

Imiqualines emerged as promising anticancer agents against hematological malignancies, substantiated by their potent efficacy against AML [29,32], adult T-cell leukemia [31], and chronic myeloid leukemia [33]. In AML, EAPB0503 exclusively targeted mutated *NPM1*, inducing selective growth arrest and apoptosis in *NPM1c* AML cells in vitro [32] and *NPM1c* AML patient blasts ex vivo [29], along with a remarkable therapeutic efficacy in treated xenograft mice with mutated *NPM1* [29,32]. Recently, a second-generation series of imiqualines was synthesized based on chemical modulation of the first-generation derivatives. EAPB02303 was identified as a lead molecule from the second generation with impressive antitumoral activity and a seemingly different mechanism of action [35]. In this study, we showed that EAPB02303 potently inhibits cell growth of both wt-*NPM1* and *NPM1c* AML cells in vitro and primary blasts from AML patients ex vivo, broadening the effect of this compound beyond our reported anti-NPM1c AML activity for EAPB0503 [29,32]. Notably, an impressively low concentration of 5 nM inhibited AML cell viability and was even more pronounced than cytarabine, the backbone of AML induction chemotherapy. These results are consistent with earlier studies that emphasize the outstanding effectiveness of EAPB02303 at low doses [35]. Our prior research showed that EAPB0503 induces sub-G0 accumulation and apoptosis only in *NPM1c* AML cells by activating caspase and P53 signaling [32]. In line with these findings, we demonstrated that EAPB02303 results in sub-G0 arrest without affecting the other cell cycle phases. Moreover, EAPB02303 triggers P53-mediated apoptosis accompanied by caspase and PARP cleavage. However, in contrast to EAPB0503, EAPB02303 induces growth arrest and apoptosis in both *NPM1c* and wt-*NPM1* AML cell lines. These results further cement our initial cell proliferation data and highlight the broader potency of EAPB02303 against different subtypes of AML at concentrations 200-fold lower than EAPB0503.

Since the effectiveness of EAPB02303 encompassed different AML cells, regardless of the *NPM1* mutation, we dissected the molecular pathways implicated in its mode of action. Recently, we unraveled that EAPB02303 exerts dual downregulation of the activity of PI3K/AKT and RAS/MAPK signaling pathways in the model organism *Caenorhabditis elegans (C. elegans)* [41]. In humans, hyperactivation of the PI3K/AKT or RAS/MAPK pathways results in uncontrollable cell growth, consequently leading to tumorigenesis [43]. In our latest report, we showed that EAPB02303 treatment decreases PI3K/AKT signaling, resulting in significant lifespan extension of *C. elegans* [41]. This was accompanied by potent suppression of a hyperactivated RAS-associated phenotype in *C. elegans*, thereby suggesting that the remarkable anticancer activity of EAPB02303 in human cells is conferred by simultaneous reduction in PI3K/AKT and RAS/MAPK molecular cascades. The PI3K/AKT/mTOR signaling pathway regulates hematopoietic cell proliferation and differentiation [42]. Notably, the constitutive activation of this pathway is essential for AML cell survival [13,17] and is associated with poor overall survival [14,15,16,17,18]. In accordance with our recent findings [41], we showed that EAPB02303 treatment decreases the levels of AKT and mTOR in AML cell lines. This was accompanied by a decrease in RAS/MAPK signaling activity, as confirmed by the reduction in the levels of the downstream effector kinase ERK. The current study therefore corroborates our previous findings and affirms that the anticancer activity of EAPB02303 is mediated through downregulation of PI3K/AKT/mTOR and RAS/MAPK signaling.

Prior evidence suggests that mTOR activity is tightly coupled to SENP3/NPM1 interplay, whereby mTOR-mediated phosphorylation of SENP3 facilitates its interaction with NPM1 [44]. NPM1 is a downstream effector of mTOR signaling, thus contributing to the deleterious cell proliferation in certain types of solid tumors [45]. Herein, the EAPB02303-mediated mTOR downregulation was paralleled by a decrease in the NPM1c levels in the *NPM1*-mutant AML cell line. This was coupled with low SENP3 and high ARF levels, consistent with our previous data in *NPM1c* AML [29]. Our results suggest that EAPB02303-mediated inhibition of the mTOR pathway triggers the nucleolar release of SENP3, likely compromising its implication in AML ribosomal biogenesis. This notion could be further supported by the increased ARF expression. Our data implies that treatment with EAPB02303 upregulates ARF to induce NPM1 SUMOylation, thereby antagonizing the NPM1-mediated ribosomal biogenesis in *NPM1c*-AML, necessitating future investigations.

We have previously established the selective in vivo efficacy of EAPB0503 on *NPM1c* AML xenograft survival, organ infiltration, and leukemia burden in the BM [29,32]. In the current study, we expanded our results to wt-*NPM1* AML xenograft mice treated with EAPB02303. Indeed, EAPB02303 significantly prolongs the survival of *NPM1c* AML xenografts for up to 120 days (*p* = 0.0009). This proves that EAPB02303 is even more potent in vivo than its analog EAPB0503, which extended the survival of treated xenograft mice to 100 days (*p* = 0.0036) [29]. To our surprise, wt-*NPM1* AML xenograft mice treated with EAPB02303 succumbed on the same day as the untreated controls. As opposed to the *NPM1c*-exclusive EAPB0503 [29], the second-generation leader EAPB02303 improved the general wellness of both *NPM1c-* and wt-*NPM1* AML-treated animals. This was evident by the significant reduction in leukemic burden in the BM, the decrease in spleen weight in all treated mice, and the drastic resolution of AML cells infiltrating the livers and the spleens upon treatment. The improved overall health of all EAPB02303-treated animals but the exclusive survival extension in *NPM1c* AML xenografts could be explained by superior treatment response conferred via the *NPM1* mutation. This idea was outlined in previous studies, which established that *NPM1* mutational status improves sensitivity to chemotherapeutical agents [46]. Our results presumably delineate that while EAPB02303 displays an NPM1-independent mechanism of action, the favorable survival outcome is facilitated by the presence of mutated *NPM1*. Altogether, our study highlights the broader potency of the new imiqualine EAPB02303 against *NPM1c* and *wt-NPM1* AML.

## 5. Conclusions

To conclude, our study provides promising AML therapeutic interventions using the novel second-generation imiqualine EAPB02303. Particularly, our results widen the scope of imiqualine-based AML treatment by presenting an original mechanism of action of EAPB02303 independently from the *NPM1* mutational status.

## Figures and Tables

**Table 1:** AML patients’ detailed information.

	AGE	GENDER	KARYOTYPE	Disease	NPM1 STATUS	NGS
**P1**	71	Female	46,XX (11)	AML	NPM1c	DNMT3A, IDH2, KDM6A, TET2, FLT3, NOTCH1
**P2**	19	Male	46,XY, t(15;17)(q24;q21)(2)/46, XY (7)	APL	WT-NPM1	N/A
**P3**	34	Male	49, XY, +8, +9,inv(16)(p13.1q22), +22[cp9]/46, XY (1)	AML	WT-NPM1	N/A
**P4**	72	Female	46, XX, del(7)(?p12)del(7)(?q31)(15)/46, XX (5)	AML	WT-NPM1	DNMT3A, IDH2, TET2, EZH2
**P5**	35	Male	46XY (20)	AML	WT-NPM1	IKZF1, PTPN1, RUNX1
**P6**	54	Female	46XX (35)	AML	NPM1c	DNMT3A, GATA1, NPM1, FLT3

**Table 2:** List of primary and secondary antibodies.

Primary Antibodies		Dilution	Species	Company
p53 (DO-1)	sc-126	1:200	Mouse	Santa Cruz Biotechnology, Dallas, TX, USA
Phospho-p53 (Ser15)	#9284	1:500	Rabbit	Cell Signaling Technology, Danvers, MA, USA
p21 Waf1/Cip1 (12D1)	#9247	1:500	Rabbit	Cell Signaling Technology
PARP-1 (F-2)	sc-8007	1:500	Mouse	Santa Cruz Biotechnology
Caspase-3 (31A1067)	sc-56053	1:500	Mouse	Santa Cruz Biotechnology
Akt (pan) (C67E7)	#4691	1:500	Rabbit	Cell Signaling Technology
Phospho-Akt (Ser473)	#9271	1:250	Rabbit	Cell Signaling Technology
mTOR (7C10)	#2983	1:500	Rabbit	Cell Signaling Technology
Phospho-mTOR (Ser2448)	#2971	1:500	Rabbit	Cell Signaling Technology
p44/42 MAPK (Erk1/2) (137F5)	#4695	1:500	Rabbit	Cell Signaling Technology
Phospho-p44/42 MAPK (Erk1/2) (Thr202/Tyr204)	#4370	1:500	Rabbit	Cell Signaling Technology
Anti-nucleophosmin antibody [3A9F1]	ab86712	1:1000	Mouse	Abcam, Cambridge, UK
NPM1 (mutant)	PA1-46356	1:1000	Rabbit	Invitrogen, Thermo Fischer Scientific, Waltham, MA, USA
SENP3	ab124790	1:500	Rabbit	Abcam
ARF	ab185620	1:500	Rabbit	Abcam
Histone (H3)	ab1791	1:10,000	Rabbit	Abcam
Horseradish peroxidase (HRP)-conjugated secondary antibodies				
mouse anti-rabbit IgG-HRP	sc-2357	1:5000		Santa Cruz Biotechnology
m-IgGK BP-HRP	sc-516102	1:5000		Santa Cruz Biotechnology

## Data Availability

Original data are available upon request to the first and corresponding authors.

## Supplementary Materials

The following supporting information can be downloaded at: https://www.mdpi.com/article/10.3390/biom15050741/s1. Table S1: Statistical analysis of AML cell proliferation using different concentrations of drugs and at various time points. Two-way ANOVA was performed to validate significance as compared to untreated control: ns (non-significant), * (*p*-value ≤ 0.05), ** (*p*-value ≤ 0.01) and *** (*p*-value ≤ 0.001); Figure S1: EAPB02303 significantly inhibits MOLM-13 cellular viability in vitro; Figure S2: Cell cycle analysis and annexin V/PI analysis of AML cell lines following treatment with EAPB02303; Figure S3: Western blot analysis of apoptotic protein expression levels in AML cell lines following treatment with EAPB02303; Figure S4: Western blot analysis of PI3K/AKT/mTOR protein expression levels in AML cell lines following treatment with EAPB02303; Figure S5: Densitometry analysis of NPM1, SENP3 and ARF protein expression levels in AML cell lines following treatment with EAPB02303. And Western blot original images.
