# Supplementary material for: The Novel Imiqualine EAPB02303 Is a Potent Drug for Treating Acute Myeloid Leukemia
Source: Biomolecules. 2025 May 20;15(5):741. doi: 10.3390/biom15050741 (PMC12109066; doi:10.3390/biom15050741)

Figure S2C

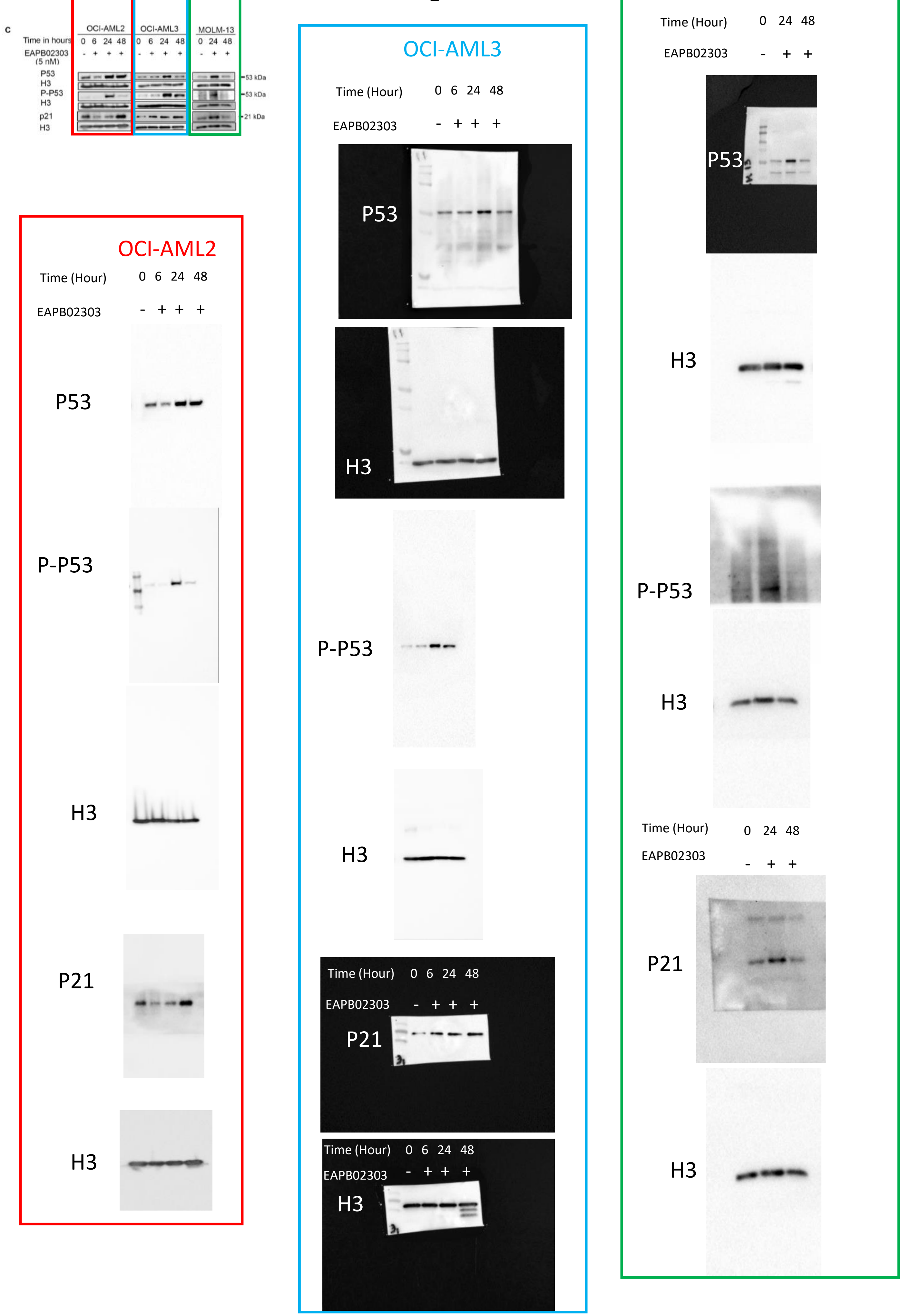
c
Time in hours
EAPB02303 (5 nM)
OCI-AML2
OCI-AML3
MOLM-13
P53
H3
P-P53
p21
53 kDa
21 kDa
OCI-AML2
Time (Hour) 0 6 24 48
EAPB02303 - + + +
P53
P-P53
H3
P21
H3
OCI-AML3
Time (Hour) 0 6 24 48
EAPB02303 - + + +
P53
H3
P-P53
H3
Time (Hour) 0 6 24 48
EAPB02303 - + + +
P21
Time (Hour) 0 6 24 48
EAPB02303 - + + +
H3
MOLM-13
Time (Hour) 0 24 48
EAPB02303 - + +
P53
H3
P-P53
H3
Time (Hour) 0 24 48
EAPB02303 - + +
P21
H3

Figure S2D

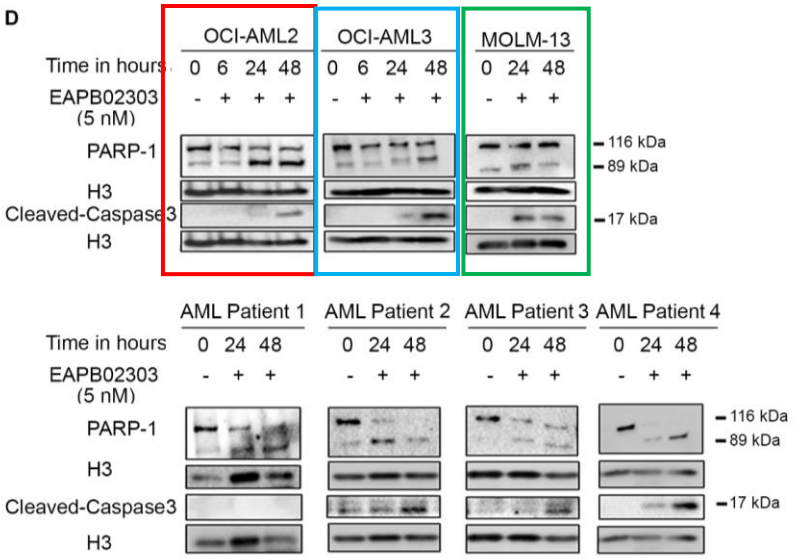
D
OCI-AML2
OCI-AML3
MOLM-13
Time in hours
0 6 24 48
0 6 24 48
0 24 48
EAPB02303
(5 nM)
- + + +
- + + +
- + +
PARP-1
H3
Cleaved-Caspase3
H3
116 kDa
89 kDa
17 kDa
AML Patient 1
AML Patient 2
AML Patient 3
AML Patient 4
Time in hours
0 24 48
EAPB02303
(5 nM)
- + +
PARP-1
H3
Cleaved-Caspase3
H3
116 kDa
89 kDa
17 kDa

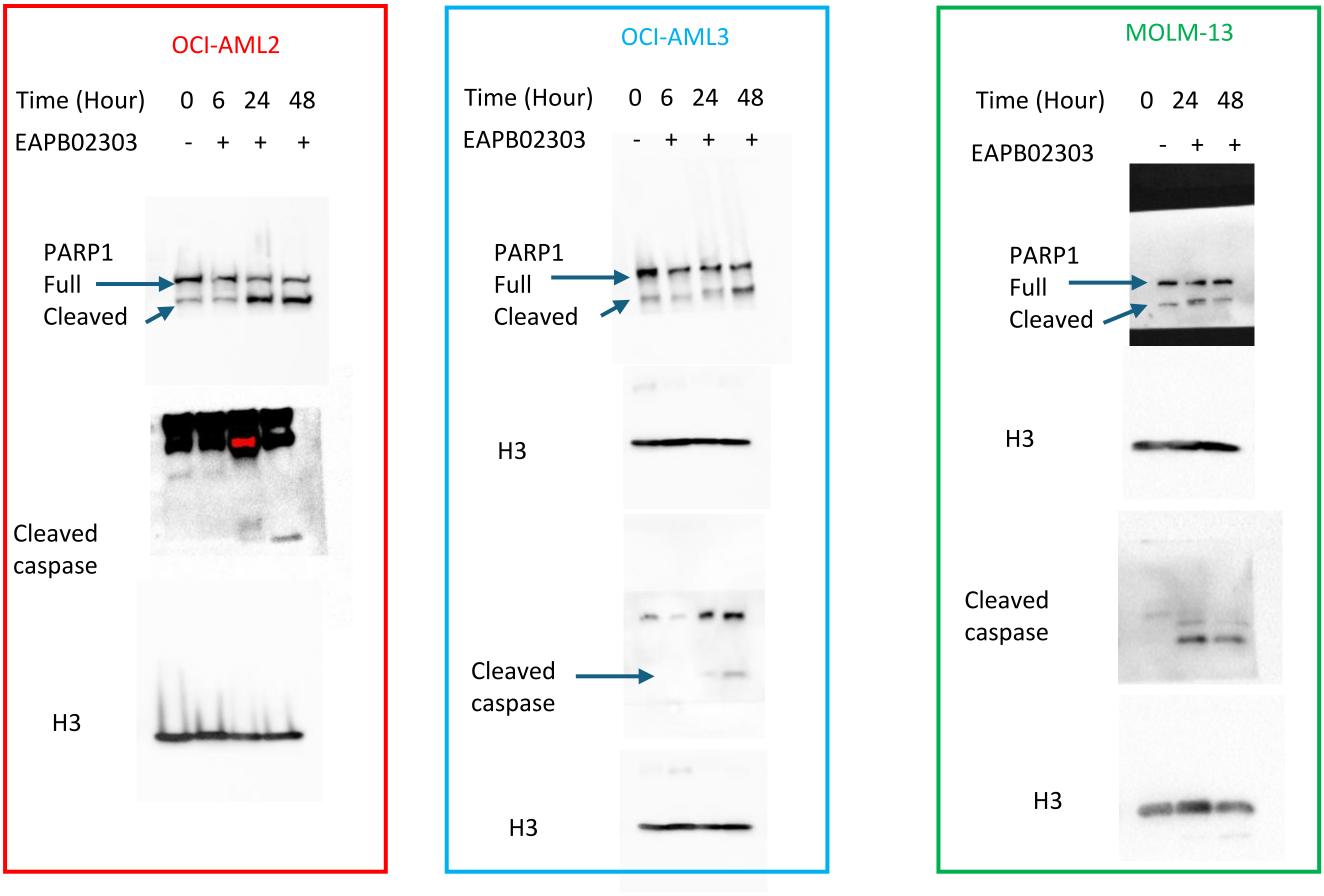
OCI-AML2
Time (Hour) 0 6 24 48
EAPB02303 - + + +
PARP1
Full
Cleaved
Cleaved
caspase
H3
OCI-AML3
Time (Hour) 0 6 24 48
EAPB02303 - + + +
PARP1
Full
Cleaved
H3
Cleaved
caspase
H3
MOLM-13
Time (Hour) 0 24 48
EAPB02303 - + +
PARP1
Full
Cleaved
H3
Cleaved
caspase
H3

Figure S2D

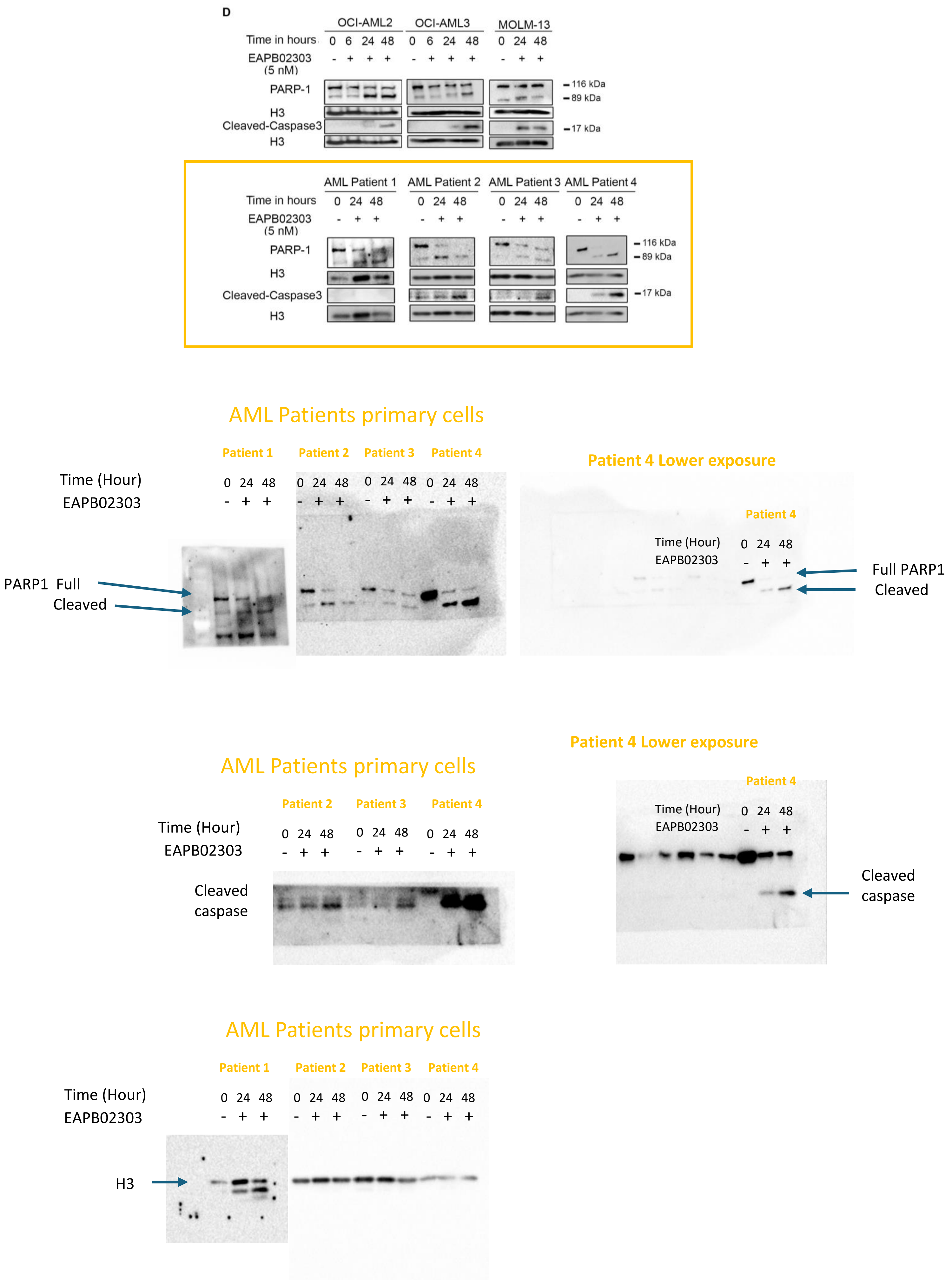
D
OCI-AML2
OCI-AML3
MOLM-13
Time in hours
0 6 24 48
0 6 24 48
0 24 48
EAPB02303 (5 nM)
- + + +
- + + +
- + +
PARP-1
H3
Cleaved-Caspase3
H3
116 kDa
89 kDa
17 kDa
AML Patient 1
AML Patient 2
AML Patient 3
AML Patient 4
Time in hours
0 24 48
EAPB02303 (5 nM)
- + +
PARP-1
H3
Cleaved-Caspase3
H3
116 kDa
89 kDa
17 kDa
AML Patients primary cells
Patient 1
Patient 2
Patient 3
Patient 4
Time (Hour)
EAPB02303
0 24 48
- + +
PARP1 Full
Cleaved
Patient 4 Lower exposure
Patient 4
Time (Hour)
EAPB02303
0 24 48
- + +
Full PARP1
Cleaved
Patient 4 Lower exposure
AML Patients primary cells
Patient 2
Patient 3
Patient 4
Time (Hour)
EAPB02303
0 24 48
- + +
Cleaved caspase
Patient 4
Time (Hour)
EAPB02303
0 24 48
- + +
Cleaved caspase
AML Patients primary cells
Patient 1
Patient 2
Patient 3
Patient 4
Time (Hour)
EAPB02303
0 24 48
- + +
H3

Figure S3A

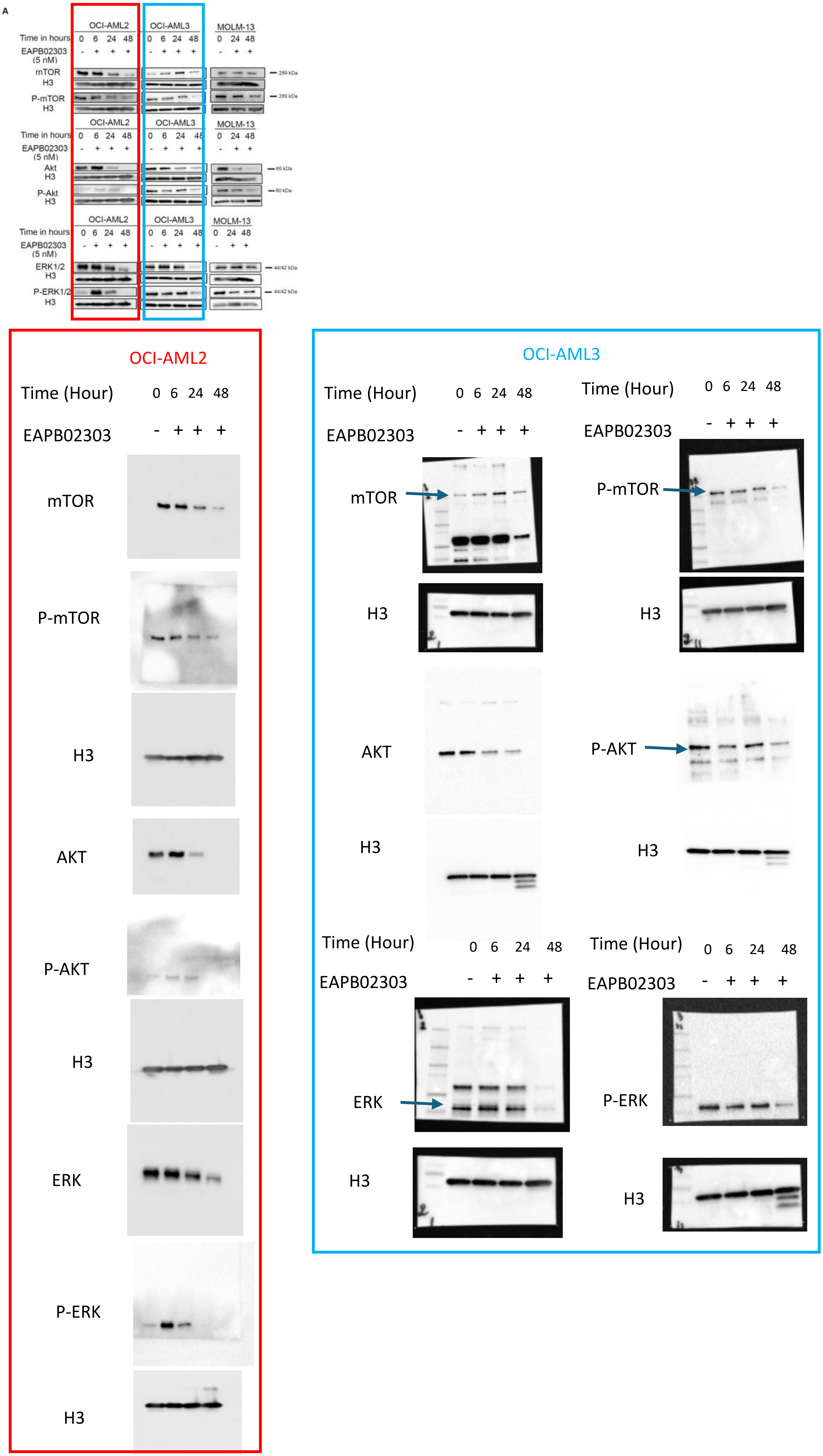

A
OCI-AML2
OCI-AML3
MOLM-13
Time in hours
EAPB02303 (5 nM)
mTOR
H3
P-mTOR
Akt
P-Akt
ERK1/2
P-ERK1/2
OCI-AML2
Time (Hour) 0 6 24 48
EAPB02303 - + + +
mTOR
P-mTOR
H3
AKT
P-AKT
ERK
P-ERK
OCI-AML3
Time (Hour) 0 6 24 48
EAPB02303 - + + +
mTOR
P-mTOR
H3
AKT
P-AKT
ERK
P-ERK

Figure S3A

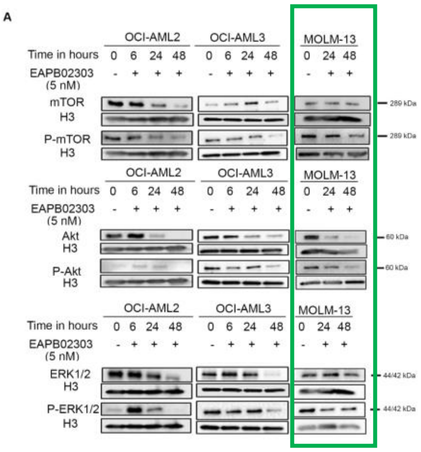

A
OCI-AML2
OCI-AML3
MOLM-13
Time in hours 0 6 24 48 0 6 24 48 0 24 48
EAPB02303 (5 nM) - + + + - + + + - + +
mTOR
H3
P-mTOR
H3
289 kDa
289 kDa
Akt
H3
P-Akt
H3
60 kDa
60 kDa
ERK1/2
H3
P-ERK1/2
H3
44/42 kDa
44/42 kDa

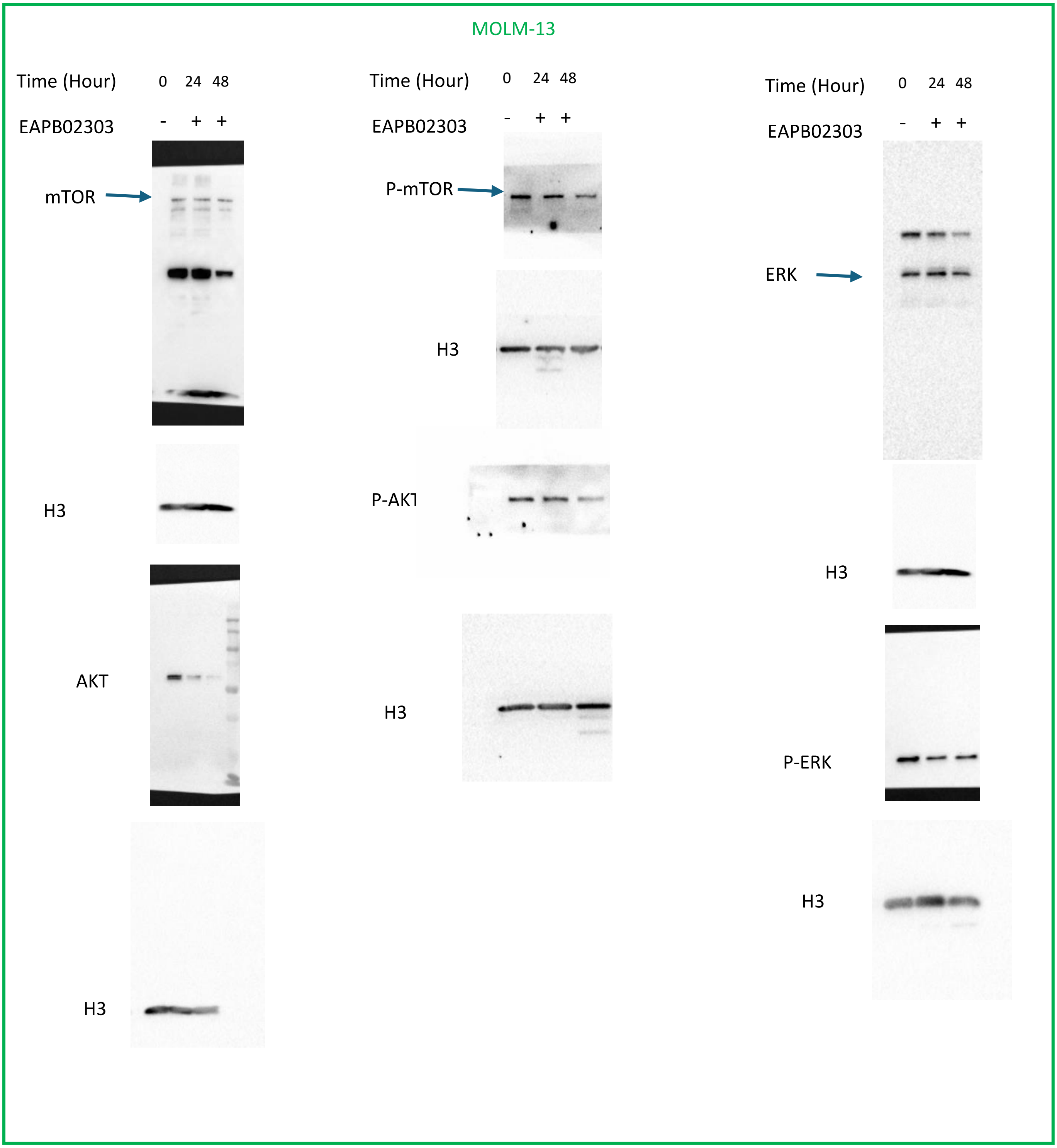

MOLM-13
Time (Hour) 0 24 48
EAPB02303 - + +
mTOR
H3
AKT
H3
Time (Hour) 0 24 48
EAPB02303 - + +
P-mTOR
H3
P-AKT
H3
Time (Hour) 0 24 48
EAPB02303 - + +
ERK
H3
P-ERK
H3

Figure S3B

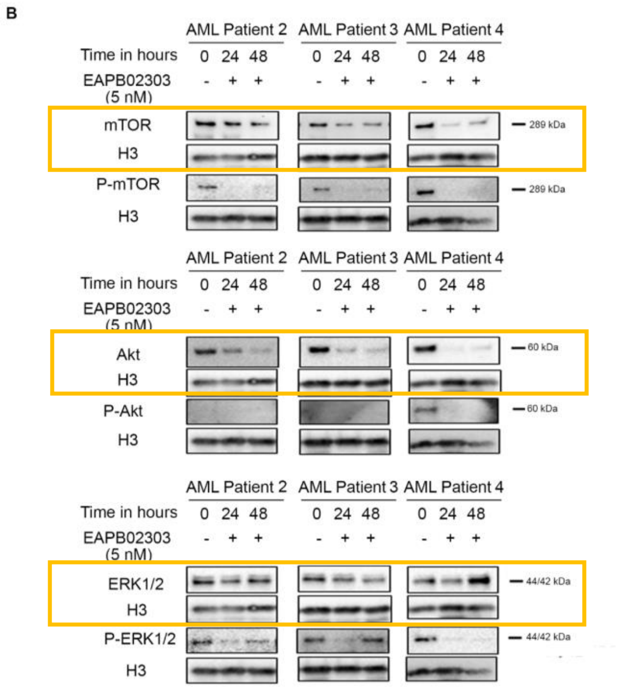
B
AML Patient 2
AML Patient 3
AML Patient 4
Time in hours
EAPB02303 (5 nM)
mTOR
H3
P-mTOR
Akt
P-Akt
ERK1/2
P-ERK1/2
289 kDa
60 kDa
44/42 kDa

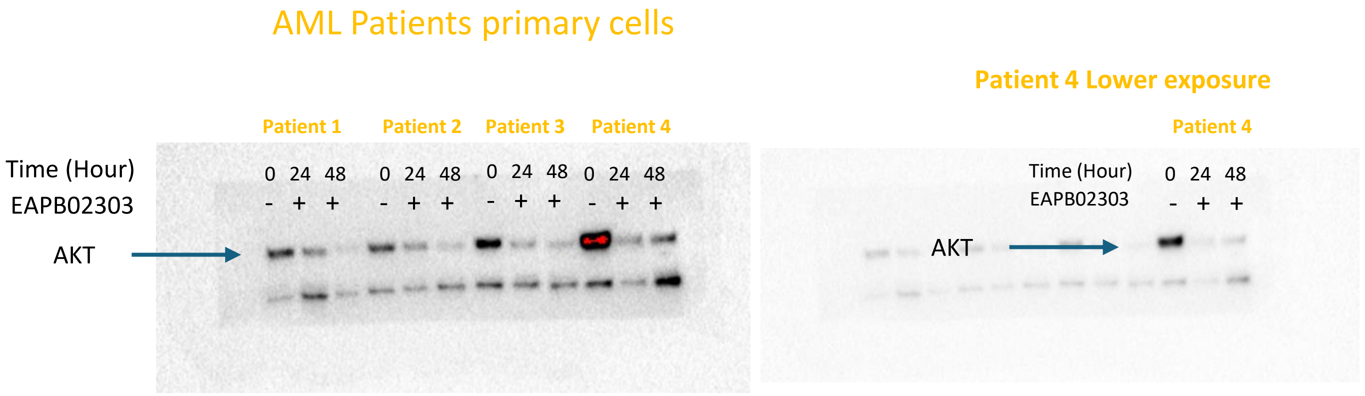
AML Patients primary cells
Patient 1
Patient 2
Patient 3
Patient 4
Time (Hour)
EAPB02303
AKT
Patient 4 Lower exposure
Patient 4
Time (Hour)
EAPB02303
AKT

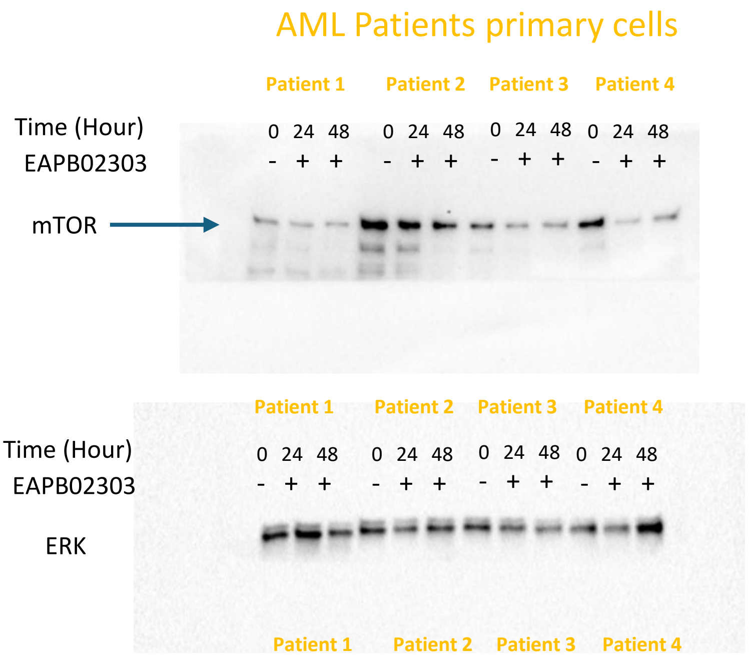
AML Patients primary cells
Patient 1
Patient 2
Patient 3
Patient 4
Time (Hour)
EAPB02303
mTOR
ERK

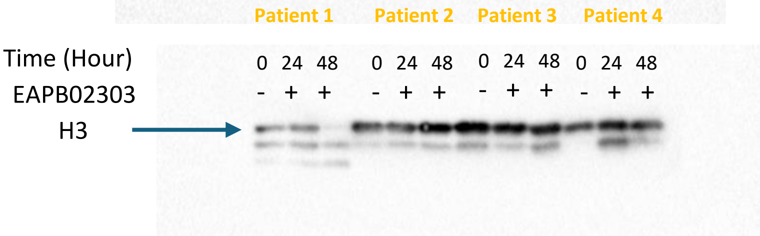
Patient 1
Patient 2
Patient 3
Patient 4
Time (Hour)
EAPB02303
H3

Figure S3B

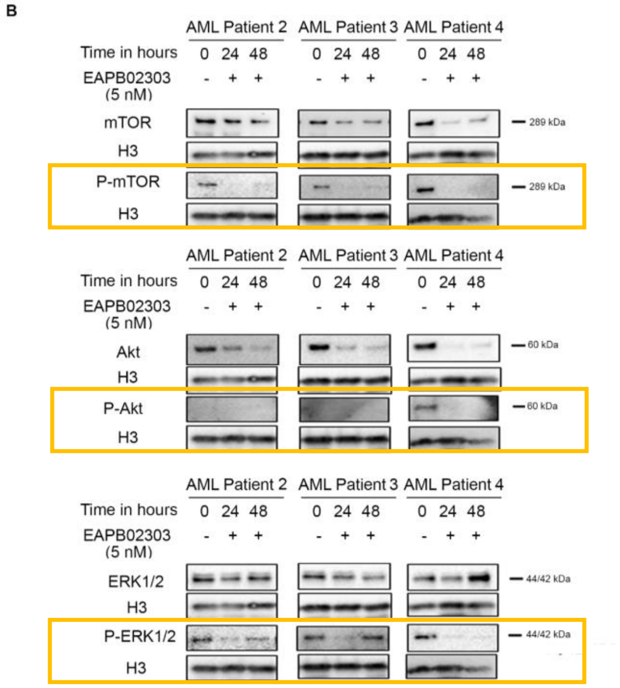

B
AML Patient 2
AML Patient 3
AML Patient 4
Time in hours
0 24 48
EAPB02303 (5 nM)
- + +
mTOR
H3
P-mTOR
289 kDa
Akt
P-Akt
60 kDa
ERK1/2
P-ERK1/2
44/42 kDa

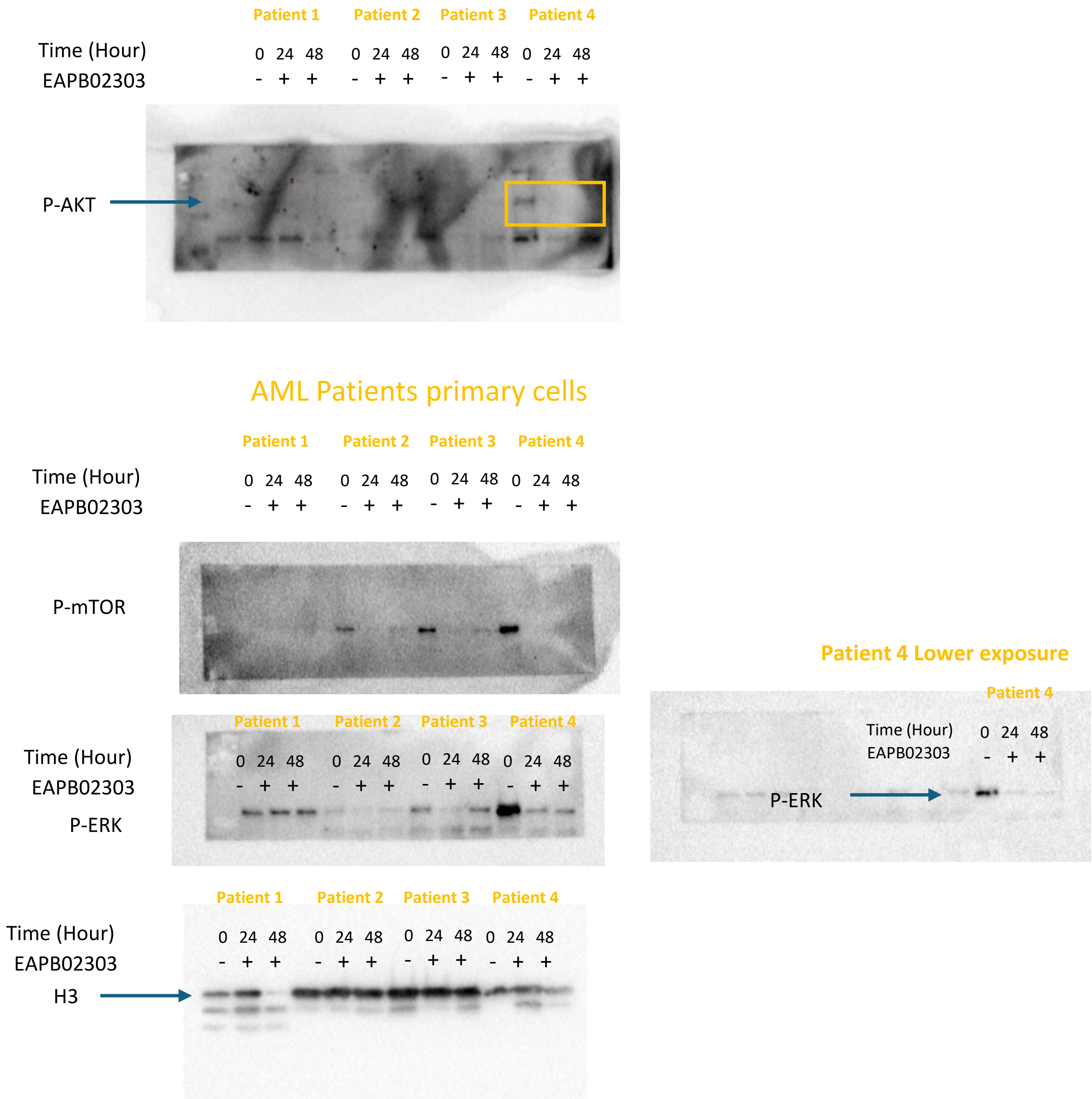

Patient 1
Patient 2
Patient 3
Patient 4
Time (Hour)
EAPB02303
0 24 48
- + +
P-AKT
AML Patients primary cells
P-mTOR
P-ERK
Patient 4 Lower exposure
H3

Figure S4

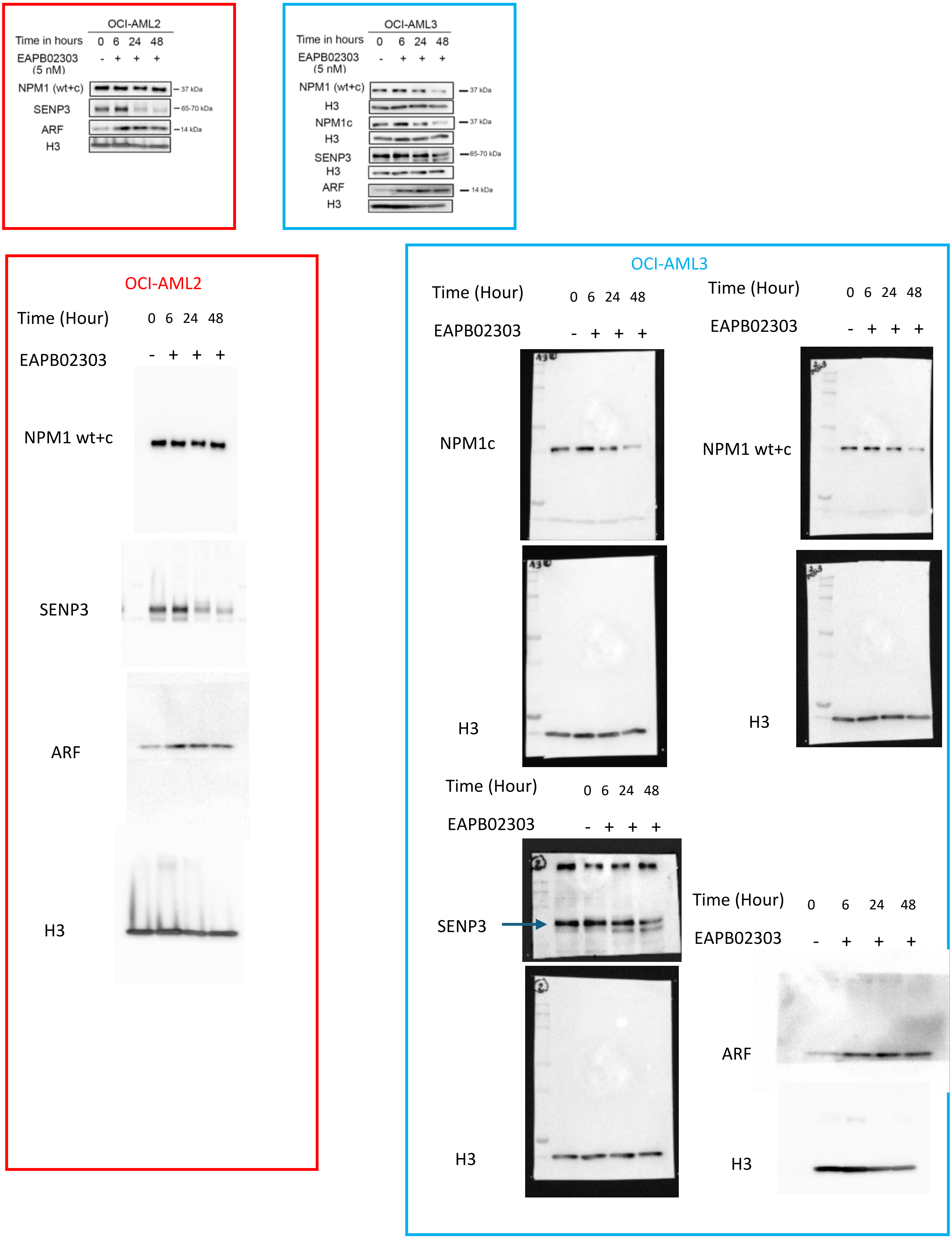

OCI-AML2
Time in hours 0 6 24 48
EAPB02303 (5 nM) - + + +
NPM1 (wt+c)
37 kDa
SENP3
65-70 kDa
ARF
14 kDa
H3
OCI-AML3
Time in hours 0 6 24 48
EAPB02303 (5 nM) - + + +
NPM1 (wt+c)
37 kDa
H3
NPM1c
37 kDa
H3
SENP3
65-70 kDa
H3
ARF
14 kDa
H3
OCI-AML2
Time (Hour) 0 6 24 48
EAPB02303 - + + +
NPM1 wt+c
SENP3
ARF
H3
OCI-AML3
Time (Hour) 0 6 24 48
EAPB02303 - + + +
NPM1c
H3
Time (Hour) 0 6 24 48
EAPB02303 - + + +
NPM1 wt+c
H3
Time (Hour) 0 6 24 48
EAPB02303 - + + +
SENP3
H3
Time (Hour) 0 6 24 48
EAPB02303 - + + +
ARF
H3

Supplementary Figure S3C

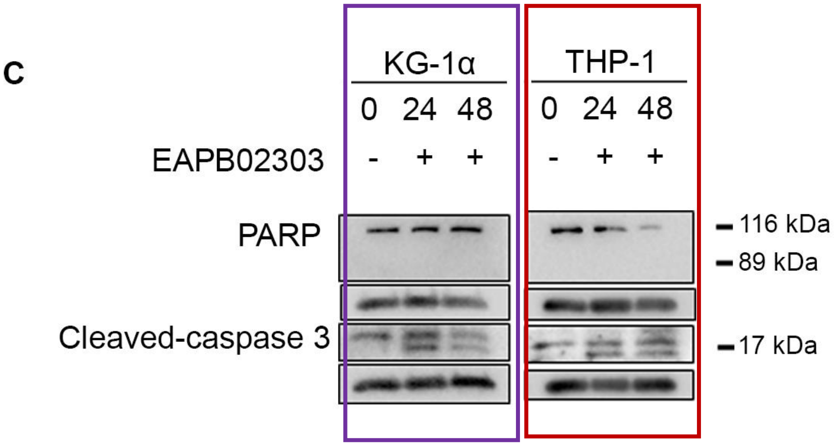

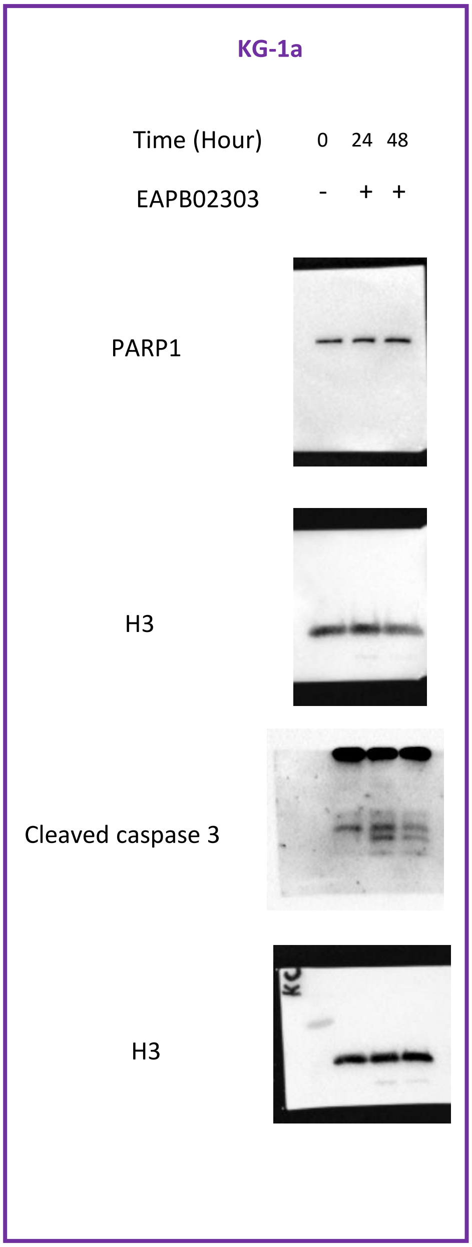

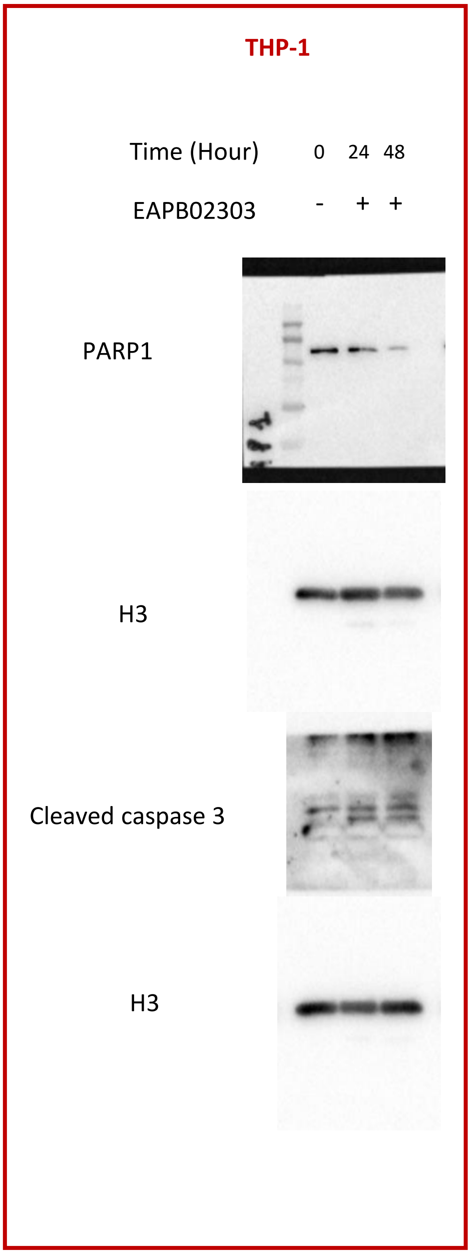

Supplementary Figure S4A

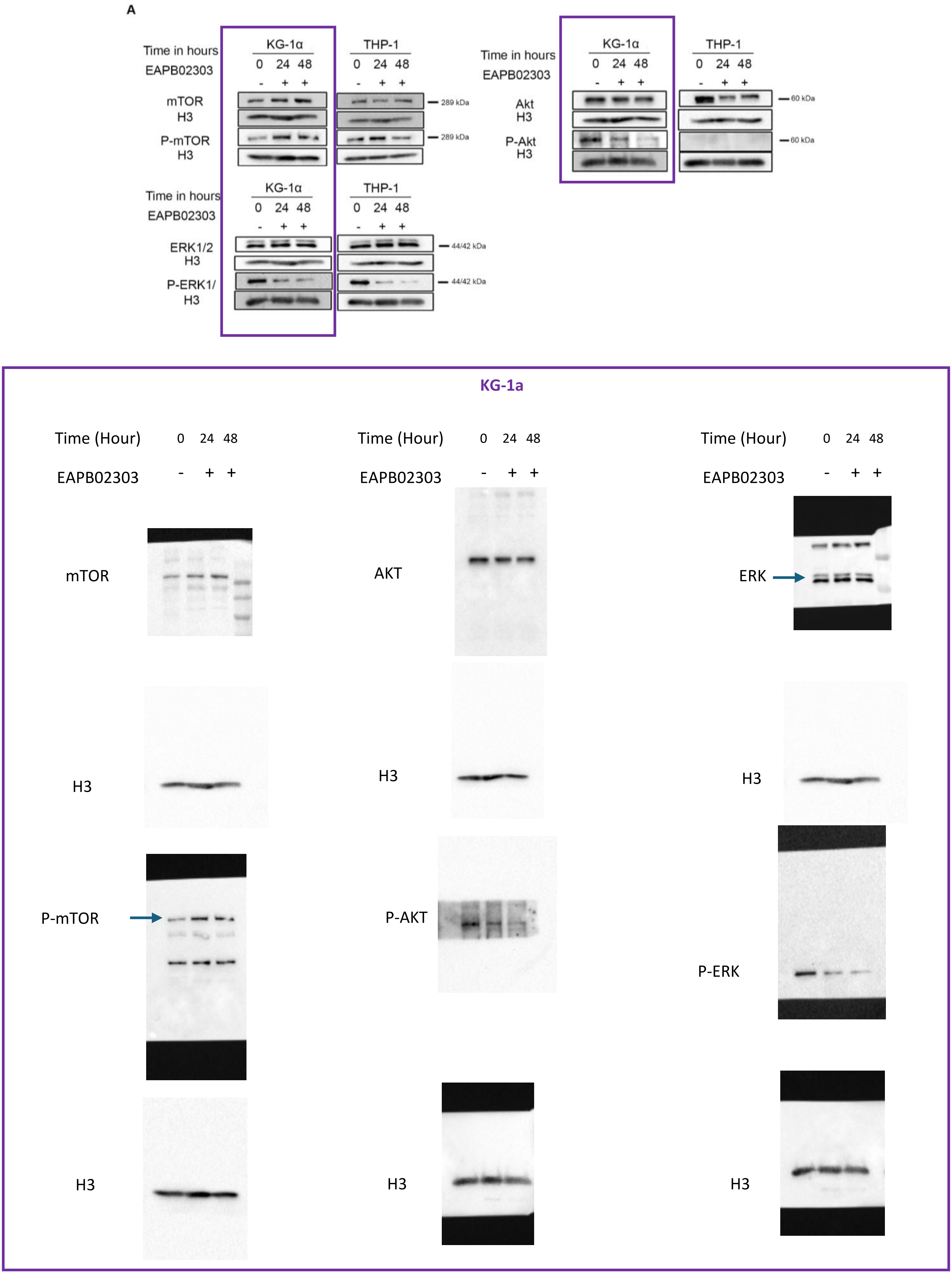

Supplementary Figure S4A

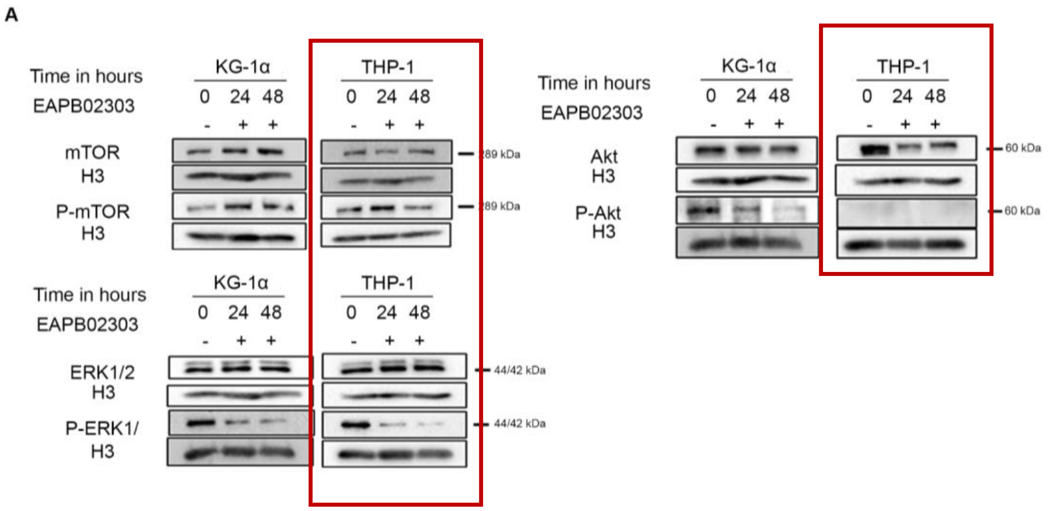

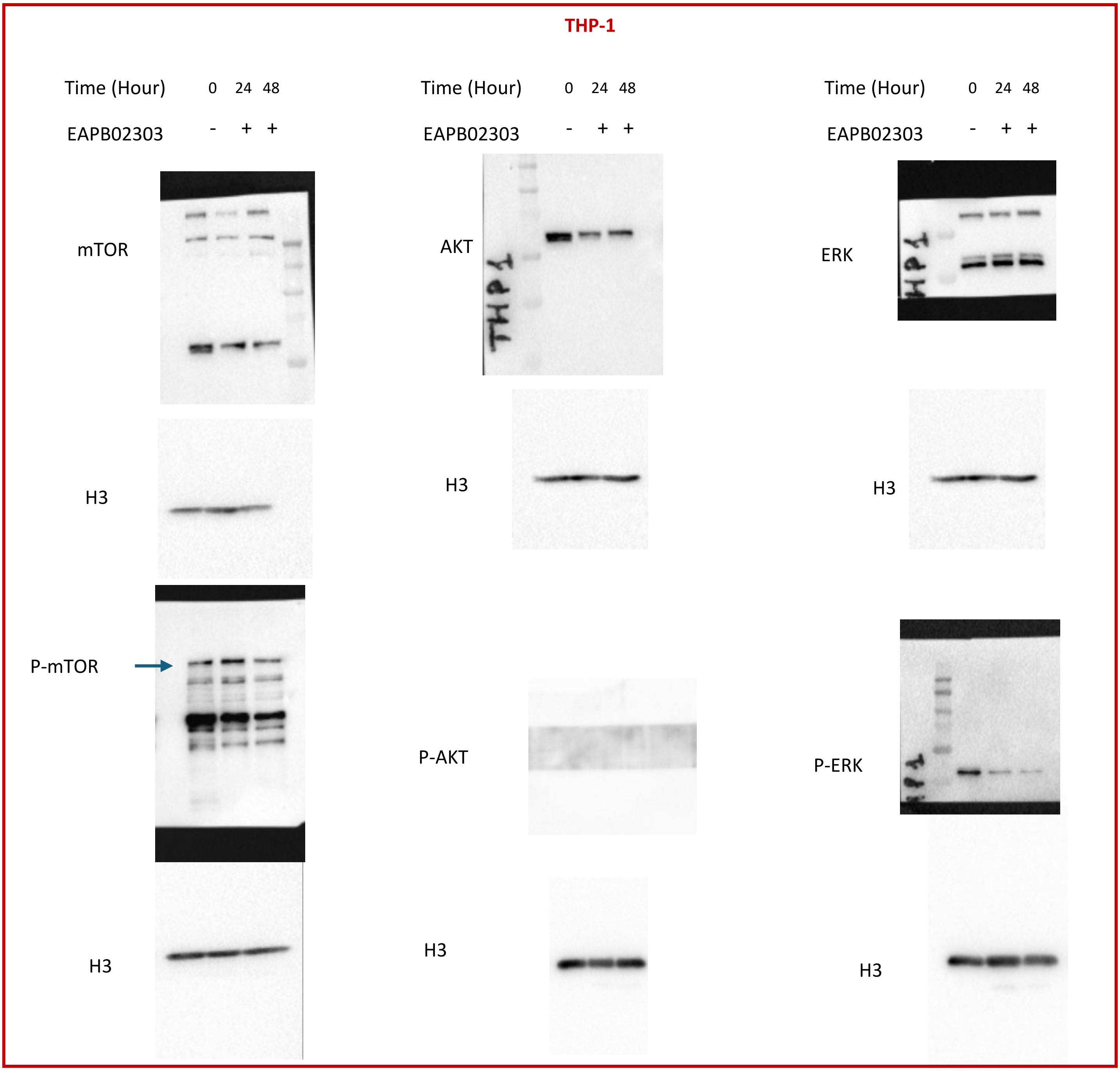

Supplement: Supplementary file 1 [file biomolecules-15-00741-s001.zip › biomolecules-3576956-original-images.pdf]
