# Supplementary material for: The Novel Imiqualine EAPB02303 Is a Potent Drug for Treating Acute Myeloid Leukemia
Source: Biomolecules. 2025 May 20;15(5):741. doi: 10.3390/biom15050741 (PMC12109066; doi:10.3390/biom15050741)
Supplement: Supplementary file 1 [file biomolecules-15-00741-s001.zip › biomolecules-3576956-supplementary.pdf]

Supplementary material

Supplementary Table S1. List of antibodies.

**Primary antibodies:**		**Company**
**p53 (DO-1)**	sc-126	Santa Cruz Biotechnology
**Phospho-p53 (Ser15)**	#9284	Cell Signaling Technology
**p21 Waf1/Cip1 (12D1)**	#9247	Cell Signaling Technology
**PARP-1 (F-2)**	sc-8007	Santa Cruz Biotechnology
**Caspase-3 (31A1067)**	sc-56053	Santa Cruz Biotechnology
**Akt (pan) (C67E7)**	#4691	Cell Signaling Technology,
**Phospho-Akt (Ser473)**	#9271	Cell Signaling Technology,
**mTOR (7C10)**	#2983	Cell Signaling Technology
**Phospho-mTOR (Ser2448)**	#2971	Cell Signaling Technology
**p44/42 MAPK (Erk1/2) (137F5)**	#4695	Cell Signaling Technology
**Phospho-p44/42 MAPK (Erk1/2) (Thr202/Tyr204)**	#4370	Cell Signaling Technology
**Anti-Nucleophosmin antibody [3A9F1]**	ab86712	Abcam
**NPM1 (mutant)**	PA1-46356	Invitrogen, ThermoFischer Scientific
**SENP3**	ab124790	Abcam
**ARF**	ab185620	Abcam
**Histone (H3)**	ab1791	Abcam
**Horseradish peroxidase (HRP)-conjugated secondary antibodies:**		
**mouse anti-rabbit IgG-HRP**	sc-2357	Santa Cruz Biotechnology
**m-IgGK BP-HRP**	sc-516102	Santa Cruz Biotechnology

Supplementary Table S2. Statistical analysis of AML cell proliferation using different concentrations of drugs and at various time points. Two-way ANOVA was performed to validate significance as compared to untreated control: ns (non-significant), * (*p*-value ≤ 0.05), ** (*p*-value ≤ 0.01) and *** (*p*-value ≤ 0.001).

**Cell lines**	**OCI-AML2**			**OCI-AML3**			**KG-1α**			**THP-1**		
**Condition/Timepoint**	**24h**	**48h**	**72h**	**24h**	**48h**	**72h**	**24h**	**48h**	**72h**	**24h**	**48h**	**72h**
**Control vs. 0.5 nM**	ns	ns	ns	ns	ns	ns						
**Control vs. 1 nM**	ns	ns	ns	ns	ns	ns	ns	ns	ns	ns	ns	ns
**Control vs. 2.5 nM**	ns	ns	***	ns	*	***	ns	*	**	ns	ns	ns
**Control vs. 5 nM**	**	***	***	*	***	***	ns	***	***	ns	ns	ns

**Control vs. 10 nM**	***	***	***	**	***	***	*	***	***	ns	ns	*
**Control vs. 100 nM**										ns	***	***
**Control vs. 500 nM**										ns	***	***
**Control vs. 1 µM**										ns	***	***
**Control vs. AraC**	*	*	**	ns	***	***						
**Patient blasts**	**AML patient 1**			**AML patient 2**			**AML patient 3**			**AML patient 4**		
**Condition/Timepoint**	**24h**	**48h**	**72h**	**24h**	**48h**	**72h**	**24h**	**48h**	**72h**	**24h**	**48h**	**72h**
**Control vs. 1 nM**	ns	*	ns	ns	ns	*	**	**	**	ns	**	**
**Control vs. 5 nM**	***	***	***	***	***	***	***	***	***	***	***	***
**Control vs. 10 nM**	***	***	***	***	***	***	***	***	***	***	***	***
**Control vs. 100 nM**	***	***	***	***	***	***	***	***	***	***	***	***
**Control vs. 500 nM**	***	***	***	***	***	***	***	***	***	***	***	***
**Control vs. 1 µM**	***	***	***	***	***	***	***	***	***	***	***	***

	**Healthy human PBMC**		
**Condition/Timepoint**	**24h**	**48h**	**72h**
**Control vs. 1 nM**	ns	ns	ns
**Control vs. 5 nM**	ns	ns	ns
**Control vs. 10 nM**	ns	ns	ns
**Control vs. 100 nM**	ns	ns	*
**Control vs. 500 nM**	ns	ns	*
**Control vs. 1 µM**	ns	ns	ns
**Control vs. 10 µM**	ns	ns	ns

Supplementary Figure 1

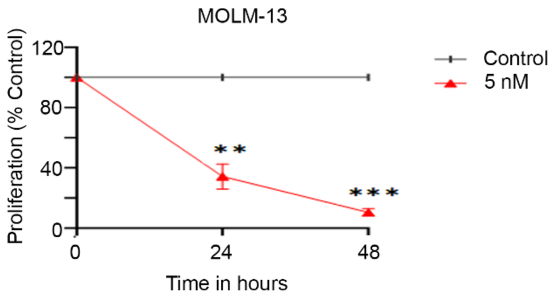

Supplementary Figure S1. EAPB02303 significantly inhibits MOLM-13 cellular viability *in vitro.* MOLM-13 cell viability was assessed using the trypan blue exclusion assay at 24h and 48h following treatment with 5 nM EAPB02303. Results shown represent the average of 3 independent experiments ± SD. Student's t-test was performed to validate significance as compared to the untreated control as follows: * *p*-value ≤ 0.05, ** *p*-value ≤ 0.01 and *** *p*-value ≤ 0.001.

Supplementary Figure 2

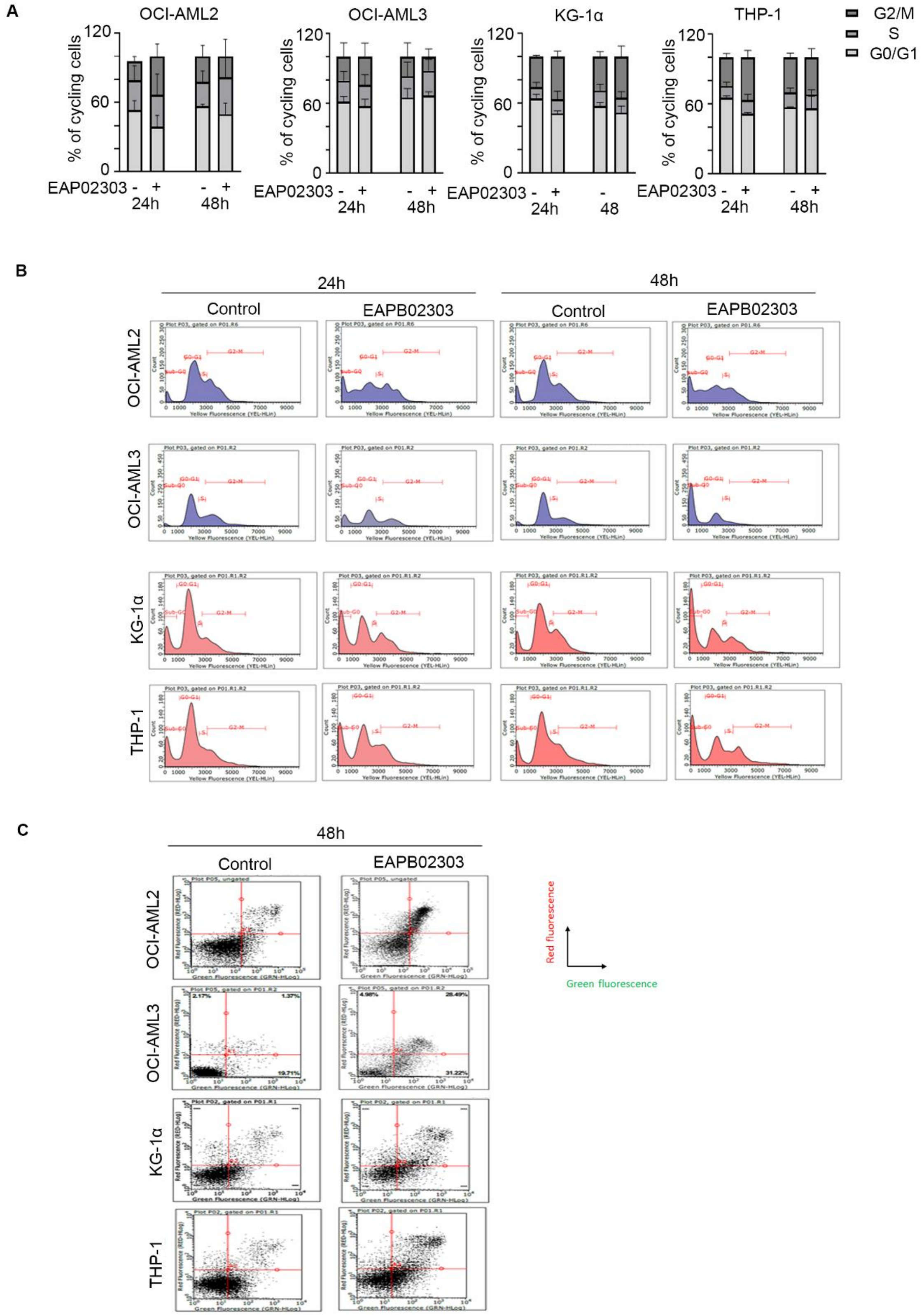

Supplementary Figure S2. Cell cycle analysis and annexin V/PI analysis of AML cell lines following treatment with EAPB02303. (A) Histograms represent the relative distributions of the non-apoptotic cycling cell populations between the G0/G1, S, and G2/M. Results shown represent the average of 3 independent experiments ± SD. (B) Individual flow cytometry graphs from one representative experiment of cell cycle analysis using PI staining, on AML cell lines OCI-AML2 and OCI-AML3 treated with 5 nM of EAPB02303, KG-1a and THP-1 cells treated with 10 nM and 100 nM of EAPB02303 respectively, for 24h or 48h. (C) Individual flow cytometry graphs from one representative experiment of Annexin V/PI staining of AML cell lines treated with EAPB02303 as described previously.

Supplementary Figure 3

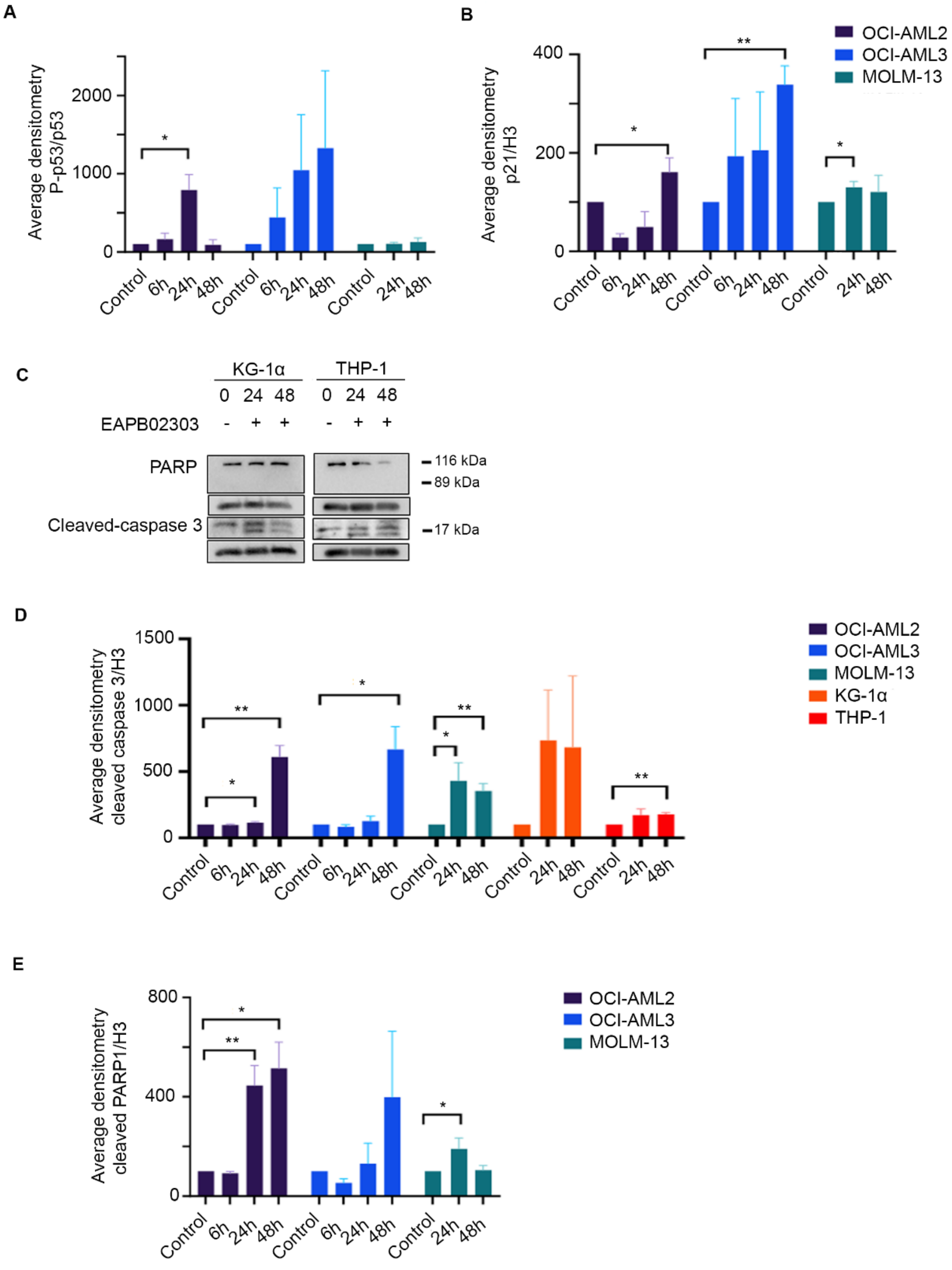

Supplementary Figure S3. Western blot analysis of apoptotic protein expression levels in AML cell lines following treatment with EAPB02303. Histograms represent the densitometry of (A) P-P53/P53 and (B) P21/H3. Densitometry was performed using ImageJ software. The results shown represent the average of at least 3 independent experiments ± SD. Student's t-test was performed to validate significance as compared to the untreated control as follows: * *p*-value ≤ 0.05, ** *p*-value ≤ 0.01 and *** *p*-value ≤ 0.001. (C) Western blot analysis of PARP-1 and cleaved caspase 3 in KG-1α and THP-1 cells treated with 10 nM or 100 nM EAPB02303, respectively. Histograms represent the densitometry of (D) cleaved caspase 3/H3 and (E) cleaved PARP1/H3. Densitometry was performed using ImageJ software. The results shown represent the average of at least 3 independent experiments ± SD. Student's t-test was performed to validate significance as compared to the untreated control as follows: * *p*-value ≤ 0.05, ** *p*-value ≤ 0.01 and *** *p*-value ≤ 0.001.

Supplementary Figure 4

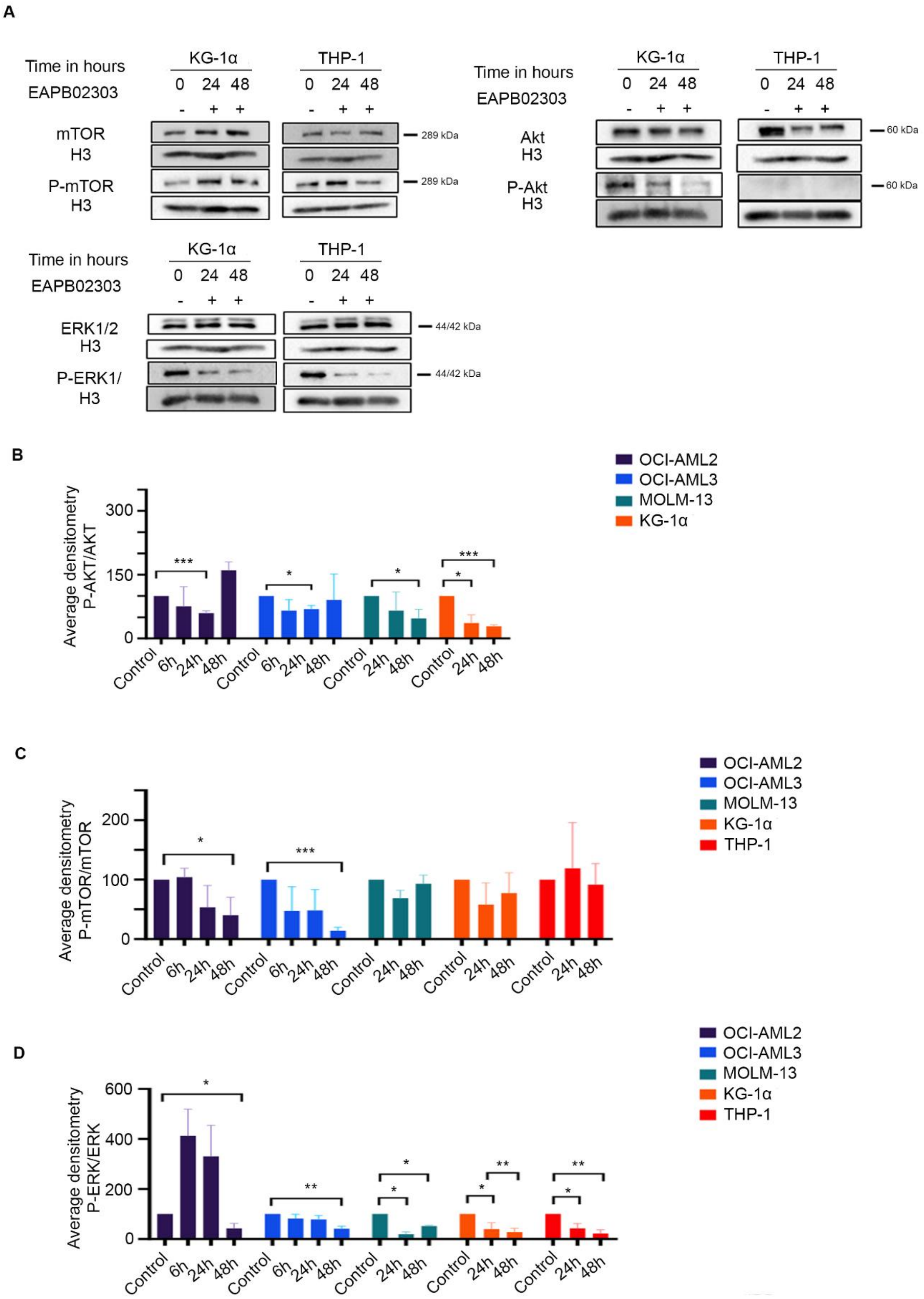

Supplementary Figure S4. Western blot analysis of PI3K/AKT/mTOR protein expression levels in AML cell lines following treatment with EAPB02303. (A) Western blot analysis of mTOR, p-mTOR, AKT, p-AKT, ERK, p-ERK and H3 in KG-1α and THP-1 cells treated with 10 nM or 100 nM EAPB02303 respectively. Histograms represent the densitometry of (B) P-AKT/AKT, (C) P-mTOR/mTOR, and (D) P-ERK/ERK. Densitometry was performed using ImageJ software. The results shown represent the average of at least 3 independent experiments ± SD. Student's t-test was performed to validate significance as compared to the untreated control as follows: * p-value ≤ 0.05, ** p-value ≤ 0.01 and *** p-value ≤ 0.001.

Supplementary Figure 5

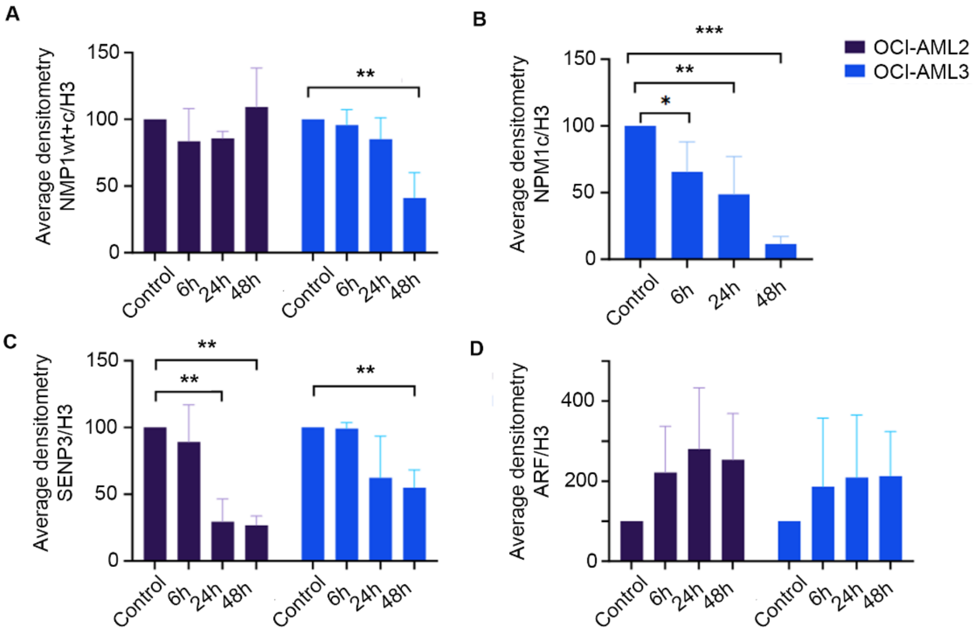

Supplementary Figure S5. Densitometry analysis of NPM1, SENP3 and ARF protein expression levels in AML cell lines following treatment with EAPB02303. Histograms represent the densitometry of (A) NPM1 (wt+c)/H3, (B) NPM1c/H3, (C) SENP3/H3, and (D) ARF/H3. Densitometry was performed using ImageJ software. The results shown represent the average of at least 3 independent experiments ± SD. Student's t-test was performed to validate significance as compared to the untreated control as follows: * *p*-value ≤ 0.05, ** *p*-value ≤ 0.01 and *** *p*-value ≤ 0.001.
